# Lycodine-Type Alkaloids from *Lycopodiastrum casuarinoides* and Their Acetylcholinesterase Inhibitory Activity

**DOI:** 10.3390/molecules19079999

**Published:** 2014-07-10

**Authors:** Dong-Bo Zhang, Jian-Jun Chen, Qiu-Yan Song, Li Zhang, Kun Gao

**Affiliations:** 1 State Key Laboratory of Applied Organic Chemistry, College of Chemistry and Chemical Engineering, Lanzhou University, Lanzhou 730000, China; 2 School of Biotechnology and Chemical Engineering, Ningbo Institute of Technology, Zhejiang University; Ningbo 315100, China

**Keywords:** *Lycopodiastrum casuarinoides*, Lycopodiaceae, lycodine alkaloid, acetylcholinesterase, 16-hydroxyhuperzine B, *N*-methyl-11-acetoxyhuperzine B, 8,15-dihydrolycoparin A, (7*S*, 12*S*, 13*R*)-huperzine D-16-*O*-β-d-glucopyranoside

## Abstract

Four new lycodine-type alkaloids, namely 16-hydroxyhuperzine B (**1**), *N*-methyl-11-acetoxyhuperzine B (**2**), 8,15-dihydrolycoparin A (**3**) and (7*S*, 12*S*, 13*R*)-huperzine D-16-*O*-β-d-glucopyranoside (**4**), along with ten known analogues **5**−**14**, were isolated from the whole plant of *Lycopodiastrum casuarinoides*. The structures of the new compounds were elucidated by means of spectroscopic techniques (IR, MS, NMR, and CD) and chemical methods. Compounds **1** and **2** possessed four connected six-membered rings, while compounds **3** and **4** were piperidine ring cleavage products. In particular, compound **4** was a lycopodium alkaloidal glycoside which is reported for the first time. Among the isolated compounds *N*-demethylhuperzinine (**7**), huperzine C (**8**), huperzine B (**9**) and lycoparin C (**13**) possessed significant inhibitory activity against acetylcholinesterase, and the new compound **1** showed moderate inhibitory activity. The structure activity relationships were discussed.

## 1. Introduction

Alzheimer’s Disease (AD) is a chronic neurological disorder characterized by memory impairment, cognitive dysfunction, behavioral disturbances and deficits in activities of daily living [1,2,3]. According to the cholinergic hypothesis, memory impairment in patients suffering from AD is a result of decreased levels of the neurotransmitter acethylcholine (ACh) in the cortex [4]. Acetylcholinesterase (AChE) inhibitors that can block the cholinergic degradation of ACh are therefore considered to be a promising approach for the treatment of AD. Additionally, recent studies have shown that AChE inhibitors also prevent the assembly of β-amyloid peptide into amyloid plaque which is the first step of AD [5,6]. This discovery further stimulated a great interest in searching for useful leads which could become new candidates for the development of rational drug design against AD.

**Figure 1:**
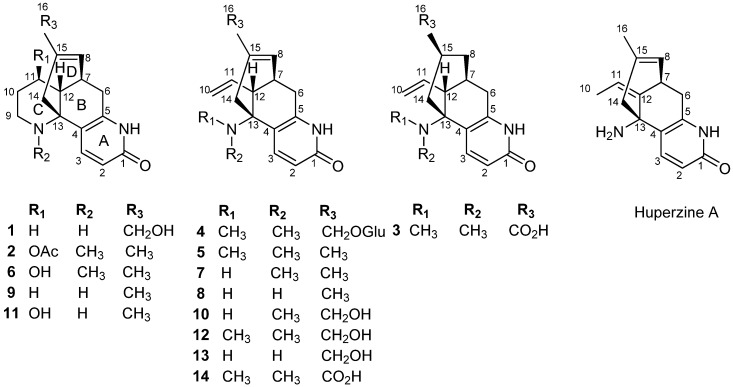
Structures of compounds **1**–**14** and huperzine A.

*Lycopodiastrum casuarinoides* (Spring) Holub (Lycopodiaceae), the only species of the genus *Lycopodiastrum*, has been used as a folk medicine for relaxing tendons and stimulating blood circulation [7]. Previous phytochemical research on this plant indicated that lycodine-type alkaloids were its main chemical and bioactive ingredients [8,9,10,11,12,13], in particular, a type of alkaloids that possessed extraordinary AChE inhibition, such as the well-known huperzine A [12]. According to previous reports and our research, it was found that the total alkaloidal extract of the plant exhibited significant AChE inhibitory activity [8,10,11,12,13]. Further bioactivity-guided chromatographic fractionation led to four new (compounds **1**−**4**) and ten known lycodine-type alkaloids **5**–**14** (Figure 1). Herein, we reported the isolation, structural elucidation, AChE inhibitory activity of these compounds. Furthermore, the preliminary structure-activity relationships are discussed.

## 2. Results and Discussion

Compound **1**, obtained as a colorless gum, showed an [M+H]^+^ ion peak at *m/z* 273.1600 in its HRESIMS, corresponding to the molecular formula of C_16_H_20_N_2_O_2_ (calcd. for C_16_H_21_N_2_O_2_, 273.1598), implying eight degrees of unsaturation. The IR absorption band (1,654 cm^−1^) of compound **1** indicated the presence of an α,β-unsaturated carbonyl group. The existence of an *α*-pyridone moiety was revealed by the absorption bands (227 and 306 nm) in its UV spectrum and two characteristic proton signals [*δ*_H_ 7.79, 6.48 (each 1H, d, *J* = 9.0 Hz)] in the low field region of the ^1^H-NMR spectrum [13]. Additionally, a hydroxymethyl group [*δ*_H_ 3.87, 3.83 (2H, ABq), *δ*_C_ 65.9] and an olefinic proton [*δ*_H_ 5.81 (br d, *J* = 4.8 Hz)] were also displayed in the ^1^H-NMR spectrum (Table 1). The ^13^C- and DEPT NMR spectra showed 16 carbon signals, including six methylenes (one oxygenated at *δ*_C_ 65.9 and one nitrided at *δ*_C_ 42.0), five methines and fivequaternary carbons (two nitrogenated at *δ*_C_ 57.6 and 145.6, and one amide carbonyl carbon at *δ*_C_ 165.5) (Table 2). These spectroscopic data of compound 1 were similar to those of the known huperzine B (9) [12] which co-occurs in this species [9], indicating that compound 1 was a lycodine alkaloid possessing four connected six-membered rings. 

**Table 1:** ^1^H-NMR data of compounds **1**–**4**, *δ* in ppm and *J* in Hz.

No.	*δ*_H_ (1) ^a^	*δ*_H_ (2) ^b^	*δ*_H_ (3) ^a^	*δ*_H_ (4) ^b^
2	6.48, d (9.0)	6.41, d (9.2)	6.46, d (9.6)	6.48, d (9.6)
3	7.79, d (9.0)	7.79, d (9.2)	7.66, d (9.6)	7.87, d (9.6)
6a	2.90, dd (18.6, 6)	2.96, dd (17.6, 4.8)	3.01, dd (18.6, 6.6)	2.99, dd (17.6, 4.3)
6b	2.39, br d (18.6)	2.39, br d (17.6)	2.36, br d (18.6)	2.38, br d (17.6)
7	2.59, m	2.68, m	2.16, m	2.60, m
8a	5.81, br d (4.8)	5.42, br d (5.2)	1.91, br d (13.8)	5.82, br d (4.1)
8b			1.78, ddd (13.8, 13.2, 3.6)	
9a	3.06, br d (12.0)	2.71, ddd (14.4, 14.2, 2.0)		
9b	2.55, overlapped	2.66, overlapped		
10a	1.80, overlapped	1.75, m	5.33, dd (16.8, 1.8)	5.42, dd (17.0, 2.0)
10b	1.80, overlapped	1.64, m	5.10, dd (10.2, 1.8)	5.27, dd (10.2, 2.0)
11a	1.39, dddd (13.2, 12.6, 12.3, 4.6)	4.76, ddd (11.2, 10.8, 5.2)	6.02, ddd (16.8, 10.2, 10.2)	6.09, ddd (17.0, 10.2, 10.1)
11b	1.69, m			
12	2.07, br d (12.6)	2.07, dd (10.8, 3.6)	2.95, dd (10.8, 3.6)	3.06, dd (10.3, 4.1)
14a	2.51, d (16.2)	2.65, d (16.7)	2.19, dd (12.6, 12.6)	3.11, d (18.4)
14b	2.25, d (16.2)	1.76, d (16.7)	1.66, dd (12.6, 3)	2.16, d (18.4)
15			2.05, m	
16	3.87, 3.83; ABq (13.8)	1.56, br s		4.11, 4.02; ABq (12.2)
Glu				
1'				4.22, d (7.8)
2'				3.17, dd (9.5, 7.8)
3'				3.23, t (9.5)
4'				3.25, t (9.5)
5'				3.34, m
6'a				3.64, dd (11.8, 5.4)
6'b				3.85, dd (11.8, 1.3)
*N*-Me _A_		2.63, s	2.58, s	2.70, s
*N*-Me _B_			2.58, s	2.70, s
11-OAc		2.04, s		

Note: **2** in CDCl_3_, **1**, **3** and **4** in CD_3_OD. ^a^ Data were measured at 600 MHz (^1^H); ^b^ Data were measured at 400 MHz (^1^H). Assignments were based on DEPT, HSQC, ^1^H-^1^H COSY, and HMBC experiments.

**Table 2:** ^13^C-NMR data of compounds **1**−**4**.

No.	*δ*_C_(1)^ a^	*δ*_C_(2) ^b^	*δ*_C_(3) ^a^	*δ*_C_(4) ^b^
1	165.5	165.0	165.0	165.5
2	119.6	118.1	118.8	118.9
3	140.6	140.8	142.4	143.1
4	115.3	119.8	117.7	117.5
5	145.6	142.6	146.4	144.9
6	29.8	29.3	30.1	29.4
7	34.3	29.7	38.7	39.7
8	127.1	124.0	37.5	128.8
9	42.0	48.6		
10	24.8	25.3	117.3	119.1
11	25.0	71.2	141.4	139.8
12	39.3	37.3	48.1	46.2
13	57.6	58.1	64.2	64.3
14	41.6	43.2	41.9	40.6
15	136.6	132.6	41.4	135.6
16	65.9	23.0	180.8	72.8
Glu				
1'				102.5
2'				75.0
3'				77.9
4'				71.7
5'				78.1
6'				62.8
*N*-Me _A_		37.2	40.2	40.2
*N*-Me _B_			40.2	40.2
11-OAc		170.5		
11-OAc		21.1		

Note: **2** in CDCl_3_, **1**, **3** and **4** in CD_3_OD. ^a^ Data were measured at 150 MHz (^13^C); ^b^ Data were measured at 100 MHz (^13^C). Assignments were based on DEPT, HSQC, ^1^H-^1^H COSY, and HMBC experiments.

**Figure 2:**
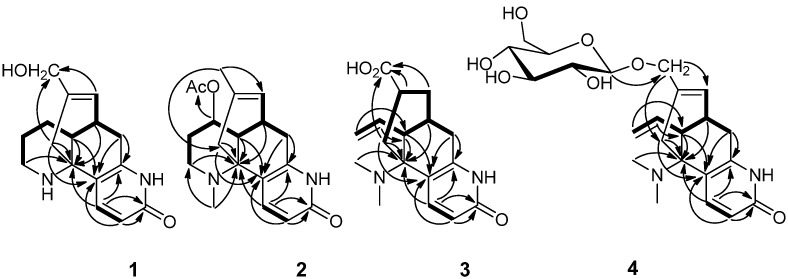
Key HMBC (H→C) correlations and ^1^H-^1^H COSY (▬) of compounds **1**–**4**.

The most significant difference between two compounds was that the presence of an additional hydroxyl group in compound **1**. The hydroxyl group might be localized to C-16, which was confirmed by HMBC correlations between the C-16 (*δ*_C_ 65.9) with H-14, H-8 (Figure 2).

**Figure 3:**
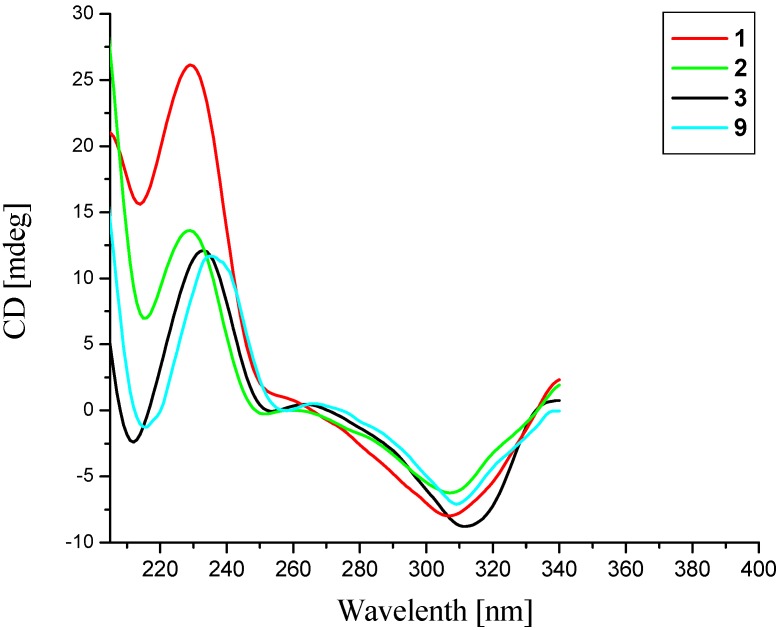
CD spectra of compounds **1**–**3** and **9**.

The relative configuration of compound **1** was provided by the NOE difference spectra. In the biogenetic consideration of lycodine-type alkaloids derivatives isolated from Lycopodiaceae species, H-12 was assigned as the β-orientation. Irradiation of H-12 enhanced the signals of H-14a, thus, the H-7 was *α*-oriented. The specific rotation of compound **1** was determined to be 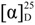 −70 (*c* 0.1, MeOH), which was similar to the value of 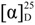 −54.2 (*c* 0.2, MeOH) observed for huperzine B (**9**) [12]. Also, the CD spectrum of compound **1** (Figure 3) showed a positive Cotton effect around 230 nm and a negative one near 307 nm, which was in agreement with that of huperzine B (**9**) [12], indicating a (13*R*) configuration [13]. Taken together, the absolute configuration of compound **1** was assigned to be 7*S*/12*R*/13*R*. Finally, the structure of compound **1** was elucidated and named as 16-hydroxyhuperzine B.

Compound **2**, obtained as a yellow powder, has an [M+H]^+^ ion peak at *m/z* 329.1863 in its HRESIMS, corresponding to the molecular formula of C_19_H_24_N_2_O_3_ (calcd. for C_19_H_25_N_2_O_3_, 329.1860), implying nine degrees of unsaturation. The NMR data of compound **2** were similar to those of huperzine B (**9**) [12], except for the presence of an additional acetoxy group [*δ*_H_ 2.04 (3H, s), *δ*_C_ 21.1, 170.5] and one methyl group [*δ*_H_ 2.63 (3H, s), *δ*_C_ 37.2]. The acetoxy group might be localized at C-11 in compound **2**, which was supported by the shift of the C-11 signal in compound **2** (*δ*_C_ 71.2) to lower field region relative to huperzine B (**9**) (*δ*_C_ 28.1), and was further confirmed by observed key HMBC correlations from H-11 (*δ*_H_ 4.76) to the acetoxy carbonyl carbon at *δ*_C_ 170.5 (Figure 2). The methyl could be attached to the *N*-atom, which was confirmed by HMBC correlations between the proton at *δ*_H_ 2.63 with C-13 (*δ*_C_ 58.1) and C-9 (*δ*_C_ 48.6) (Figure 2). The relative configuration of compound **2** was deduced by the NOE difference spectra experiment and the coupling constants. Like compound **1**, the H-12 was assigned a β-orientation and the H-7 was *α*-oriented. Furthermore, the large coupling constant between H-11 and H-12 (*J*_11, 12_ = 10.8 Hz) indicated the vicinal protons H-11 (*δ*_H_ 4.76) and H-12 (*δ* 2.07) both took axial orientations. The CD spectrum of compound **2** showed a positive Cotton effect at 229 nm and a negative one at 307 nm (Figure 3), which matched well with that of huperzine B (9) [12]. Thus, the absolute configuration of compound **2** was assigned as 7*R*/11*R*/12*R*/13*R*.

Compound **3**, a white powder, showed an [M+H]^+^ ion peak at *m/z* 303.1709 in its HRESIMS, corresponding to the molecular formula of C_17_H_22_N_2_O_3 _(calcd. for C_17_H_23_N_2_O_3_, 303.1703), implying eight degrees of unsaturation. In the ^1^H-NMR spectrum of compound **3**, an ABX spin system for the exomethylene moiety (CH_2_ = CH) resonating at *δ*_H_ 6.02 (1H, ddd, *J* = 16.8, 10.2, 10.2 Hz), 5.33 (1H, dd, *J* = 16.8, 1.8 Hz) and 5.10 (1H, dd, *J* = 10.2, 1.8 Hz) suggested that compound **3** was a piperidine ring (C ring) cleavage product. The NMR data of compound **3** were highly similar to those of lycoparin A (**14**) [8], except for the fact the Δ^8(15)^ double bond was saturated, which implied the structure of compound **3** to be 8,15-dihydrolycoparin A, which was supported by its 2D NMR experiments (Figure 2). In particular, a long spin system [−CH_2_−CH(X)−CH_2_−CH−CH_2_−(H_2_-6/H-7(X)/H_2_-8/H-15/H_2_-14), X = −CH−CH−CH_2_−(H-12/H-11/H_2_-10)] was displayed in the ^1^H-^1^H COSY spectrum of compound **3**. The relative configuration of compound **3** was deduced by the NOE difference spectra experiment and the coupling constants. In the consideration of the biogenesis of the lycodine-type alkaloid derivatives isolated from Lycopodiaceae species, the H-12 was assigned as the β-orientation. Irradiation of H-12 enhanced the signals of H-8b and H-14a, indicating that these protons were on the same facial plane, the H-7 was therefore assigned as the *α*-orientation. The large coupling constant between H-15 and H-14a (*J*_14a_, _15_ = 12.6 Hz) indicated that these two protons both took axial orientations, thus, H-15 was on the other side. Similarly, the CD curve of compound **3** showed a positive Cotton effect around 230 nm and a negative value near 310 nm (Figure 3). Consequently, the absolute configuration of compound **3** was established as 7*S*/12*R*/13*R*/15*R*.

Compound **4**, obtained as a colorless powder, showed a molecular ion peak at *m/z* 449.2288 [M+H]^+^ in HRESIMS, consistent with the molecular formula of C_23_H_32_N_2_O_7_ (calcd. for 449.2282, C_23_H_33_N_2_O_7_) requiring nine degrees of unsaturation. The strong IR absorptions at 3,385 and 1,658 cm^−1^ indicated the presence of hydroxyl and carbonyl groups, respectively. The NMR data of compound **4** were analogous to those of huperzine D (**12**) [11] with a C-15 hydroxymethyl group except for a set of signals of one hexose group including the anomeric proton signal at *δ*_H_ 4.22 (1H, d, *J* = 7.8 Hz), the other 6 proton signals at *δ*_H_ 3.17–3.85, the anomeric carbon signal at *δ*_C_ 102.5 (C-1) and another 5 carbon signals at *δ*_C_ 62–78. The hexose was suggested to be a D-glucose by the comparison of ^13^C-NMR data with those reported in the literature, and further confirmed by TLC comparison with an authentic sample and by its specific rotation value after acidic hydrolysis of compound **4** in HCl-methanol (9%) yielded huperzine D (**12**) and glucose [14]. The coupling constant of H-1' (*δ*_H_ 4.22, d, *J* = 7.8 Hz) indicated the D-glucose had a β-linkage. The C-16 of compound **4** should be glycosylated, which was confirmed by the observed key HMBC correlation from the anomeric proton at H-1' (*δ*_H_ 4.22) to C-16 (*δ*_C_ 72.8) (Figure 2). Accordingly, the structure of compound **4** was assigned as (7*S*, 12*S*, 13*R*)-huperzine D-16-O-β-D-glucopyranoside.

By comparison of our spectroscopic data with those reported in the literature, the remaining known compounds were identified as huperzinine (**5**) [11], casuarinine A (**6**) [13], *N*-demethylhuperzinine (**7**) [10], huperzine C (**8**) [11], huperzine B (**9**) [12], casuarinine E (**10**) [13], carinatumin B (**11**) [15], huperzine D (**12**) [11], lycoparin C (**13**) [8], and lycoparin A ( **14**) [8], respectively.

**Figure 4:**
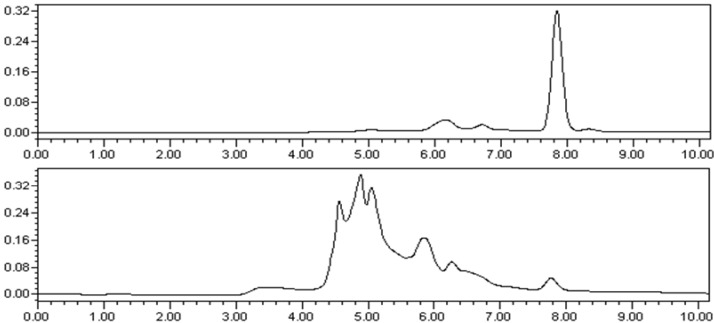
(**A**) HPLC analysis of compound **2** (retention time: 7.848 min); (**B**): HPLC analysis of the MeOH extracts of *L. casuarinoides* (retention time: 7.821 min). HPLC analyses were performed on Waters 1525-2998 series HPLC system (C-18 column, Sun Fire, 5 μm, 4.6 mm × 150 mm); mobile phase, CH_3_CN/H_2_O = 7/3; flow rate, 1.0 mL/min; injection volume, 10 μL).

Lycodine-type alkaloids are a group of structurally unique secondary metabolites, characterized by four or three connected six-membered rings, including a pyridone or pyridine ring (ring A), a piperidine ring (ring C), and a bicyclo[3.3.1]nonane core formed by rings B and D. Many of them continued to be the hot spots of research interest because of their promising bioactivity [16] and challenging total synthesis [17,18]. In our study, four new lycodine-type alkaloids and ten known analogues were isolated from *L*. *casuarinoides*. As far as we know, compound **4** is the first reported example of a lycopodium alkaloidal glycoside. It may be worthwhile to point out that we initially doubted the origin of compound **2** because it could be an artifact produced from the present extraction and isolation procedure, which employed EtOAc. In order to rule out that possibility, the MeOH extract of the plant was re-examined by the HPLC with pure compound **2** as reference (Figure 4). The unequivocal detection of the same compound in the original extract with an identical HPLC t_R_ value as that of the reference compound proved that compound **2** was a natural product and not an artifact.

The isolated lycodine-type alkaloids **1**–**14** were evaluated for their AChE inhibiting activity by Ellman’s method in 96-well microplates [19,20]. The results are listed in Table 3. Huperzine A with an IC_50_ value of 74.3 nM was used as a reference compound, and it showed good agreement with literature data (IC_50_ = 72.4 nM) [21]. Huperzine C (**8**) possessed the most potent inhibition against AChE, with an IC_50_ value of 0.6 μM. Also, *N*-demethylhuperzinine (**7**), huperzine B (**9**) and lycoparin C (**13**) showed significant AChE inhibitory activity with IC_50_ values of 1.9, 20.2 and 23.9 μM, respectively, and the new compound **1** exhibited moderate inhibitory activity with IC_50_ value of 87.3 µM. Interestingly, *N*-demethylhuperzinine (**7**), huperzine C (**8**), huperzine B (**9**) and lycoparin C (**13**) have been previously reported to show inhibition of AChE activity with IC_50_ values of 15.0, 0.489, 19.3 and 25.0 µM, repectively [8,13,21]. Our reports were found to be close to the literature data. In fact, the inhibiton of huperzine C (**8**) with an amino group at C-13 was 3-fold higher than that of *N*-demethyl-huperzinine (**7**), and huperzinine (**5**) show very weak activity, suggested that the *N*-methyl group on the position 13 might cause the sharp decrease in AChE inhibition. In other words, the amino group on the positions 13 may be a structural requirement for the anti-AChE activity of lycodine-type alkaloids, which was also supported by comparing the structure-activity relationships between casuarinine E (**10**) (IC_50_ > 250 μM) and lycoparin C (**13**). Morever, the activity of huperzine C (**8**) with a methyl at C-15 was 40-fold higher than those of lycoparin C (**13**) with a hydroxymethyl at C-15, indicated the methyl of the three-carbon bridge ring was important for AChE inhibition in this kind of alkaloids. Interestingly, huperzine A is very similar to huperzine C (**8**), except for the position of double bond, but the activity of the former was 8-fold higher than that of the latter, implying the exocyclic double bond was required for high anti-AChE activity. The findings mentioned above were consistent with previous observations [22].

**Table 3:** AChE inhibiting activity of compounds **1**, **7**, **8**, **9** and **13**.

Compounds	IC_50 _(μM)
Compound **1**	87.3 ± 1.9
*N*-Demethylhuperzinine (**7**)	1.9 ± 0.2
Huperzine C (**8**)	0.6 ± 0.1
Huperzine B (**9**)	20.2 ± 1.3
Lycoparin C (**13**)	23.9 ± 2.2
Huperzine A ^a^	(74.3 ± 2.8) ×10^−3^

Values are expressed as mean ± SD (n = 3). ^a^ Positive control.

## 3. Experimental

### 3.1. General

Optical rotations were measured on a Perkin-Elmer 341 polarimeter. IR spectra were recorded on a 170SX FT-IR instrument using KBr discs over the range of 400–4,000 cm^−1^. UV spectra were measured using a Shimadzu UV-260 spectrophotometer. CD spectra were obtained on an Olis DSM 1000 spectrometer. NMR spectra were recorded on a Bruker AM-400 and a Varian Mercury-600BB NMR (600 MHz) spectrometer using TMS as an internal standard. High-resolution electrospray ionization mass spectra (HRESIMS) were measured on a Bruker Daltonics APEX II 47e spectrometer. Column chromatography was performed on silica gel (200–300 mesh, Qingdao Marine Chemical Inc., Qingdao, People’s Republic of China), Sephadex LH-20 (GE Healthcare Bio-Sciences AB, Uppsala, Sweden) and RP-C18 (100–200 µm, Waters). Semipreparative HPLC was performed on a Waters 1525 binary pump system with a Waters 2489 detector (210 nm) using a YMC-Pack ODS-A (250 × 10 mm, S-5 μm) column. Fractions were monitored by TLC, which were visualized by heating the silica gel plates after being sprayed with 5% H_2_SO_4_ in EtOH.

### 3.2. Plant Material

The whole *L. casuarinoides* were collected in Tunchang County of Hainan Province, China (19°36′N; 110°12′E; elevation 328 m), in July 2009, and identified by Qiongxin Zhong of Hainan Normal University. The voucher specimen (No. 2009020) was deposited in the State Key Laboratory of Applied Organic Chemistry, Lanzhou University, China.

### 3.3. Extraction and Isolation

An air-dried and powdered sample (4.7 kg) was extracted with 95% MeOH three times (each time 30 L for 7 days) at room temperature. Evaporation of the solvent gave a residue (430 g), which was partitioned between EtOAc (3 × 2 L) and 2% HCl solution (2 L). The acidic water-soluble materials, adjusted to pH 9–10 with 10% ammonia solution, were extracted with CHCl_3_ (4 × 2.5 L) and BuOH (4 × 2.5 L). The CHCl_3_ extract (4.3 g) was subjected to silica gel (Φ 4 × 40, 200–300 mesh, 400 g) column eluting with a CHCl_3_/MeOH (80:0, 80:1, 40:1, 20:1, 10:1, 5:1, 2:1, 1:1, 0:1, each 1.5 L) gradient system to give fractions 1–9. Fraction 3 (0.15 g) was chromatographed on silica gel column (Φ 1 × 10, 200–300 mesh, 8 g) eluting with PE/EtOAc/Et_2_NH (1:1:0.002, 0.1 L) to give compound 2 (6.0 mg). Huperzinine (**5**, 900 mg) was recrystallized in MeOH from fraction 4 (1.4 g). Fraction 5 (0.3 g) was subjected to silica gel column chromatography (Φ 1.5 × 15, 200–300 mesh, 30 g) eluting with CHCl_3_/EtOAc/MeOH/Et_2_NH (7:7:1:0.015, 0.15 L) to yield casuarinine A (**6**) (20.6 mg), *N*-demethyl-huperzinine (**7**) (15.6 mg) and huperzine C (**8**) (10.3 mg). Fraction 6 (0.3 g) was rechromatographed on silica gel (Φ 1.5 × 15, 200–300 mesh, 30 g) eluting with EtOAc/MeOH/Et_2_NH (10:1:0.01, 0.13 L) to give huperzine B (**9**) (15.7 mg). Fraction 7 (0.5 g) was chromatographed on a silica gel column (Φ 2 × 10, 200–300 mesh, 35 g) eluted with CHCl_3_/EtOAc/MeOH, (5:5:1) to afford three fractions (7A–C). Then fraction 7B was purified by reversed-phase preparative HPLC (MeOH/H_2_O, 28:72, v/v; flow rate, 2.0 mL/min) to yield compound **3** (1.2 mg, *t*_R_ = 16.9 min), casuarinine E (**10**) (2.8 mg, *t*_R_ = 20.9 min), carinatumin B (**11**) (18 mg, *t*_R_ = 18.6 min) and huperzine D (**12**) (19.2 mg, *t*_R_ = 35.3 min). The BuOH extract (2.3 g) was subjected to an HP-20 column (Φ 5 × 20, 0.8 kg) eluting with a H_2_O/MeOH (10:0, 9:1, 8:2, 7:3, 6:4, 5:5, each 2 L) gradient system to give fractions 1–6. Fraction 2 (0.15 g) was chromatographed on a reversed-phase column (Φ 1 × 15, 30 g) using ODS and was eluted with 20% MeOH (fractions 2A–B). Then fraction 2B was purified by reversed-phase preparative HPLC (MeOH/H_2_O, 16:84, v/v; flow rate, 2.0 mL/min) to yield lycoparin C (**13**) (3.1 mg, *t*_R_ = 8.9 min), lycoparin A (**14**) (3.8 mg, *t*_R_ = 11.2 min), compound **1** (1.8 mg, *t*_R_=19.3 min) and compound **4** (6.3 mg, *t*_R_ = 12.6 min).

### 3.4. Spectral Data

*16-Hydroxyhuperzine B* (**1**). Colorless gum; 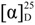 = −70 (*c* 0.1, MeOH); IR (KBr) *ν*_max_: 3340, 2919, 2850, 1654, 1611, 1464, 1115, 752 cm^−1^; UV (MeOH) λ_max_ (log *ε*): 227 (3.90), 306 (3.81) nm; ^1^H-NMR (CD_3_OD, 600 MHz) and ^13^C-NMR (CD_3_OD, 150 MHz) data: see Table 1 and Table 2; HRESIMS *m/z* 273.1600 [M+H]^+^ (273.1598 calcd. for C_16_H_21_N_2_O_2_).

*N-Methyl-11-acetoxyhuperzine B* (**2**). Yellow powder; 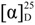 = −50 (*c* 0.1, MeOH); IR (KBr) *ν*_max_: 3373, 2919, 2850, 1658, 1598, 1456, 1095, 761 cm^−^^1^; UV (MeOH) λ_max _(log *ε*): 226 (3.86), 306 (3.79) nm; ^1^H-NMR CDCl_3_, 400 MHz) and ^13^C-NMR (CDCl_3_, 100 MHz) data: see Table 1 and Table 2; HRESIMS *m/z* 329.1863 [M+H]^+^ (329.1860 calcd. for C_19_H_25_N_2_O_3_).

*8,15-Dihydrolycoparin A* (**3**). White powder; 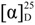 = −120 (*c* 0.1, MeOH); IR (KBr) *ν*_max_: 3384, 2919, 2851, 1658, 1609, 1462, 1119, 796 cm^−1^; UV (MeOH) λ_max _(log *ε*): 227 (3.85), 306 (3.75) nm; ^1^H-NMR (CD_3_OD, 600 MHz) and ^13^C-NMR (CD_3_OD, 150 MHz) data: see Table 1 and Table 2; HRESIMS *m/z* 303.1709 [M+H]^+^ (303.1703 calcd. for C_17_H_23_N_2_O_3_).

*(7S,12S,13R)-HuperzineD-16-O-β-d-glucopyranoside* (**4**). Colorless powder; 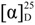 = −70 (*c* 0.2, MeOH); IR (KBr) *ν*_max_: 3385, 2919, 2851, 1658, 1602, 1459, 1093, 761 cm^−1^; UV (MeOH) λ_max _(log *ε*): 227 (3.81), 305 (3.69) nm; ^1^H-NMR (CD_3_OD, 400 MHz) and ^13^C-NMR (CD_3_OD, 100 MHz) data: see Table 1 and Table 2; HRESIMS *m/z* 449.2288 [M+H]^+^ (449.2282 calcd. for C_23_H_33_N_2_O_7_).

### 3.5. Hydrolysis of Compound **4**

Compound **4** (5 mg) was dissolved in 9% dry HCl-Methanol (2 mL) at 80 ºC for 3 h. After neutralization with NaHCO_3_, the mixture was evaporated. The residue was resuspended in H_2_O and then filtered to yield compound **1** (1.2 mg). The sugar components in the filtrate were identified by TLC (on silica gel, developed with CHCl_3_/MeOH = 5:1) as D-(+)-glucose (Rf 0.40) by comparison with an authentic sample.

### 3.6. Biological Material

AChE (EC3.1.1.7, Sigma product no. C2888), acetylthiocholine iodide (ATCI), 5,5'-dithiobis [2-nitrobenzoic acid] (DTNB) and huperzine A (purity 98%) were purchased from Sigma (St. Louis, MO, USA).

### 3.7. Assay of AChE Inhibition

The fourteen compounds were tested for AChE inhibitory activities by the modified Ellman’s method in 96-well microplates [19,20]. Briefly, 0.1 M sodium phosphate buffer (140 µL, pH = 8.0), sample solution (20 µL) and enzyme solution (15 µL) were mixed and incubated at 4 °C for 20 min. Ten µL of 0.01 M DTNB was added and the reaction was then started by adding 0.075 M ATCI (10 µL). After incubating the reaction solution at 37 °C for 20 min, the optical densities were measured in a 96-well plate reader at 405 nm immediately. A blank positive control was set up by adding 20 µL huperinze A (0.100 µg/mL in phosphate buffered saline) instead of 20 µL sample solution. Blanks were set up by adding 20 µL buffer solutions instead of 20 µL sample solution. Experiment control was set up by adding 15 µL buffer solutions instead of 15 µL enzyme solution in order to deduct the sample background. All reactions were carried out thrice. The inhibition rate (%) was calculated by the following equation:

Inhibition% = [(Blank − Blank positive control) − (Experiment − Experiment control)]/(Blank − Blank positive control) × 100%

The concentration of test samples that inhibited the hydrolysis of acetylthiocholine by 50% (IC_50_) was determined by monitoring the effect of increasing concentrations of these samples in assays on the inhibition values. Huperzine A was chosen as the positive control, with an IC_50_ value of 74 nmol.

## 4. Conclusions

A bioactivity-guided separation of the alkaloidal extracts of *L. casuarinoides* led to four new (compounds **1**–**4**) and ten known (compounds **5**–**14**) lycodine-type alkaloids. Their isolation, purification, and structural determination are reported. Within the series of lycodine-type alkaloids tested for AChE inhibiting activity, *N*-demethylhuperzinine (**7**), huperzine C (**8**), huperzine B (**9**) and lycoparin C (**13**) showed significant AChE inhibitory activity, with IC_50_ values of 1.9, 0.6, 20.2 and 23.9 μM, respectively, and the new compound **1** exhibited moderate inhibitory activity with an IC_50_ value of 87.3 μM. The structure-activity relationships disclosed that the amino group at C-13, methyl of the three-carbon bridge ring and the exocyclic double bond were all required for the anti-AChE activity of these lycodine-type alkaloids. These findings indicated that the promising AChE inhibitory activity of *N*-demethylhuperzinine (**7**), huperzine C (**8**), huperzine B (**9**) and lycoparin C (**13**) could stimulate future development of new anti-AD agents.

## Supplementary Materials

Supplementary materials can be accessed at: http://www.mdpi.com/1420-3049/19/7/9999/s1.
