# Supplementary material for: Lycodine-Type Alkaloids from Lycopodiastrum casuarinoides and Their Acetylcholinesterase Inhibitory Activity
Source: Molecules. 2014 Jul 10;19(7):9999–10010. doi: 10.3390/molecules19079999 (PMC6271639; doi:10.3390/molecules19079999)

# Supplementary Information

**Figure S1.** $^1$H-NMR spectrum of compound **1.**

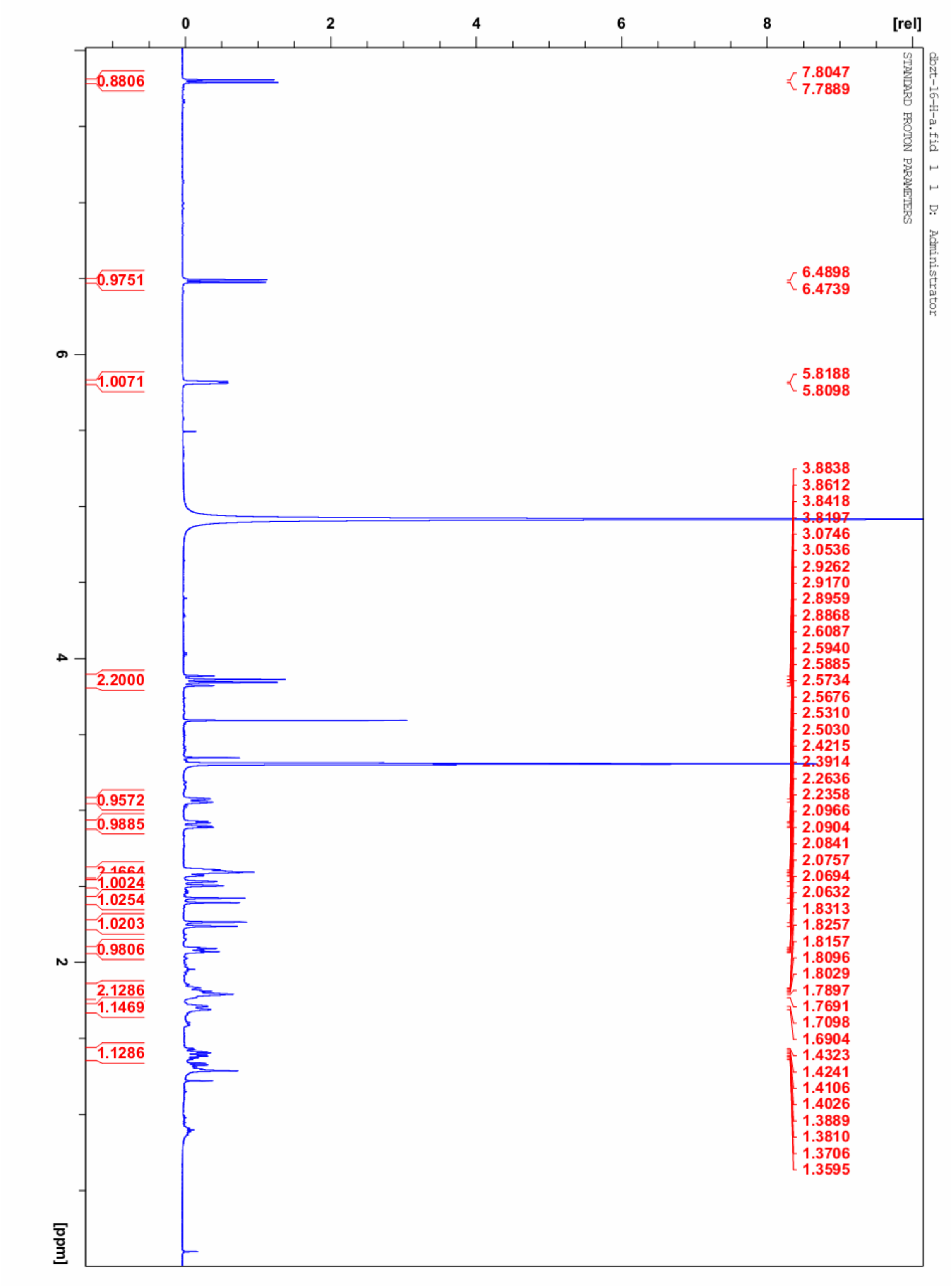

**Figure S2.** $^{13}$C-NMR spectrum of compound **1**.

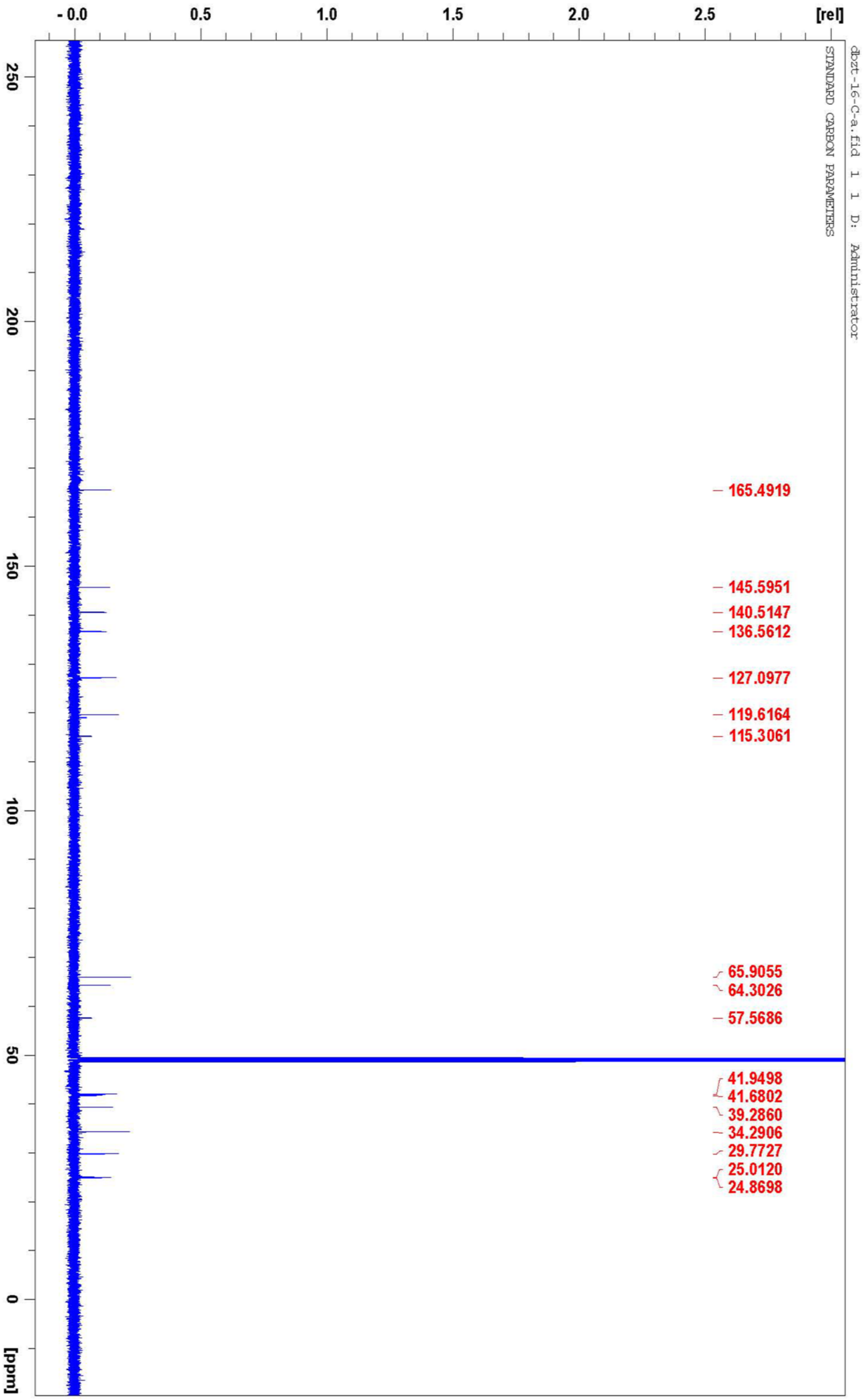

**Figure S3.** $^1$H-$^1$H COSY spectrum of compound **1**.

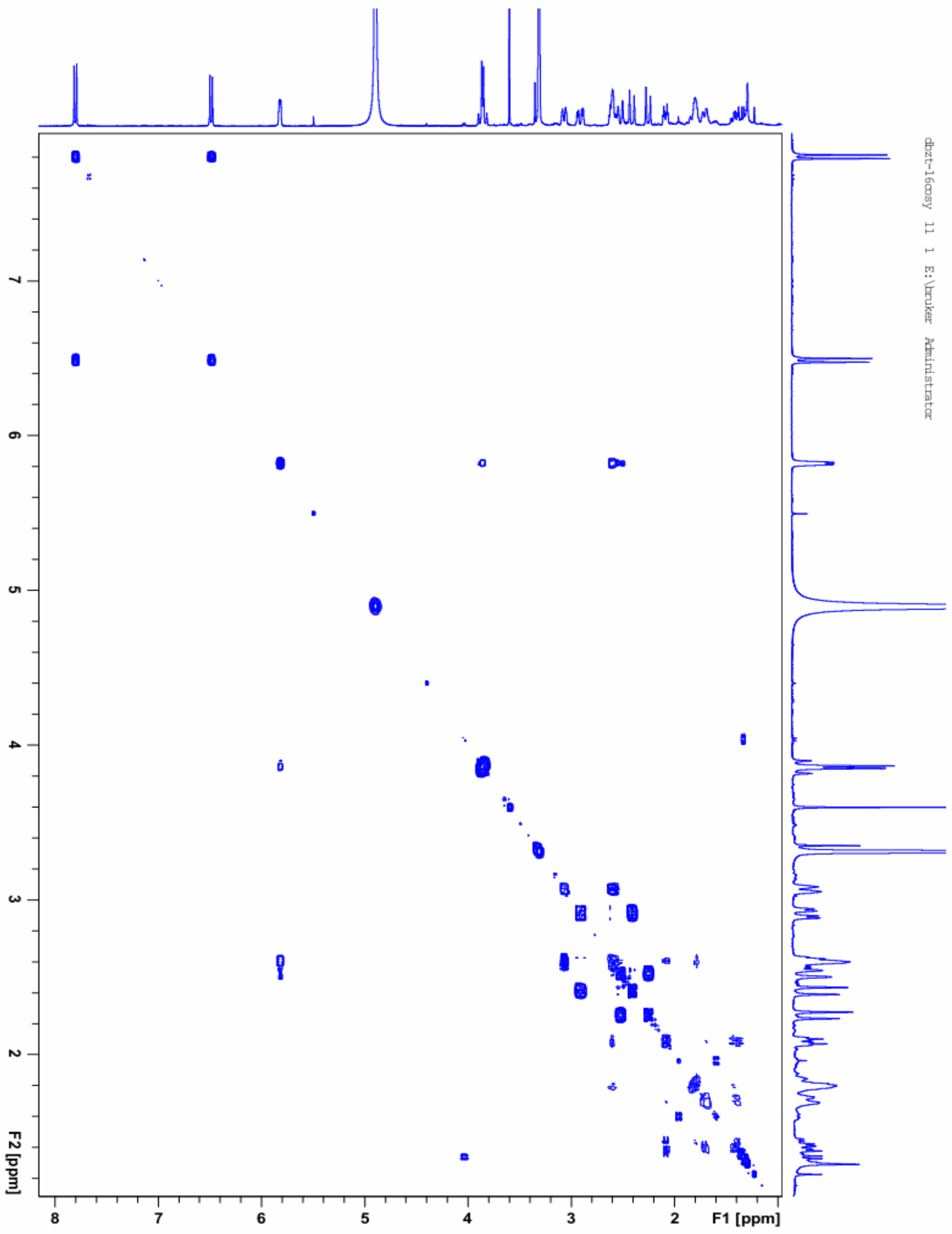

**Figure S4.** HSQC spectrum of compound **1**.

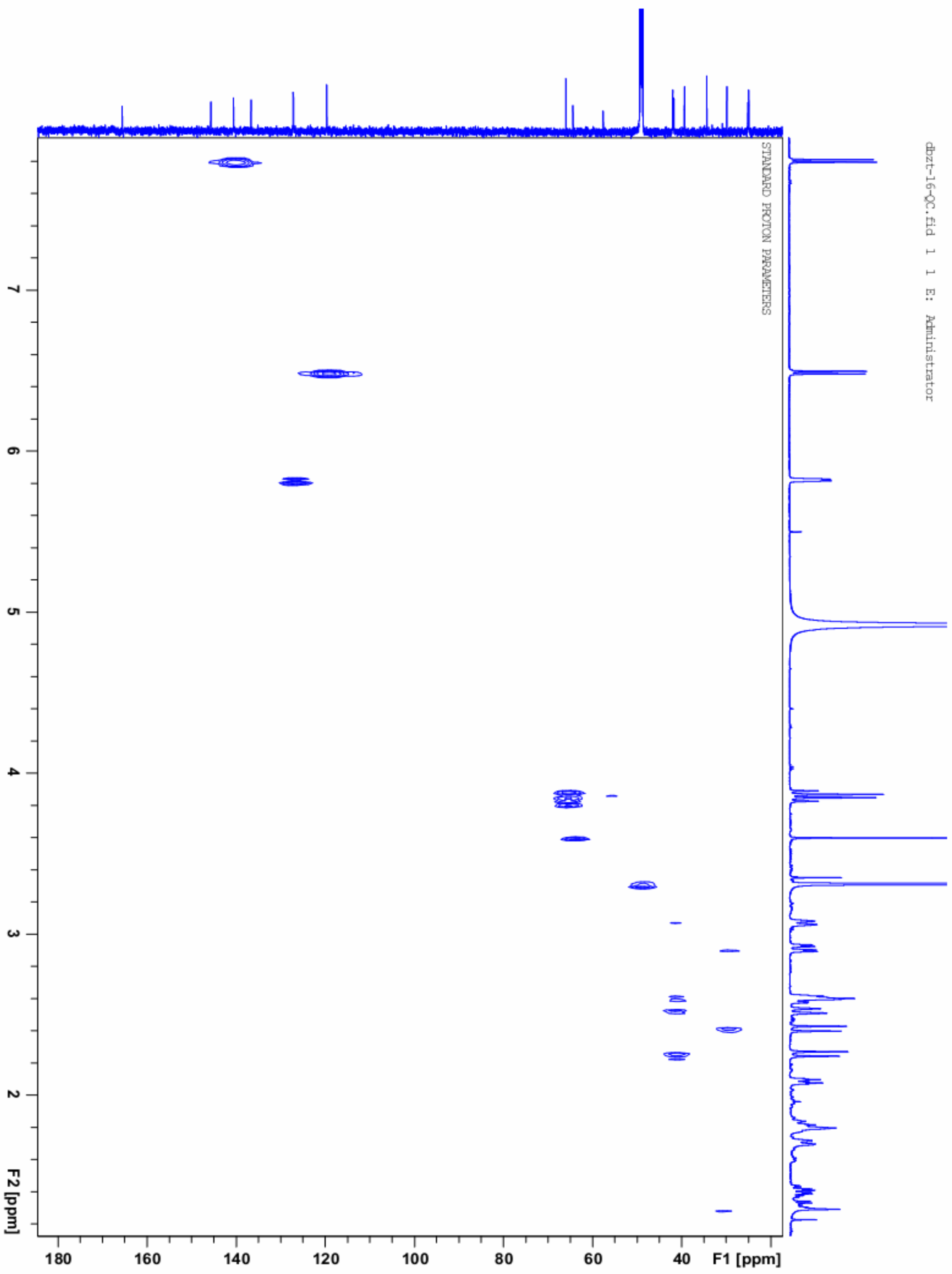

**Figure S5.** HMBC spectrum of compound **1**.

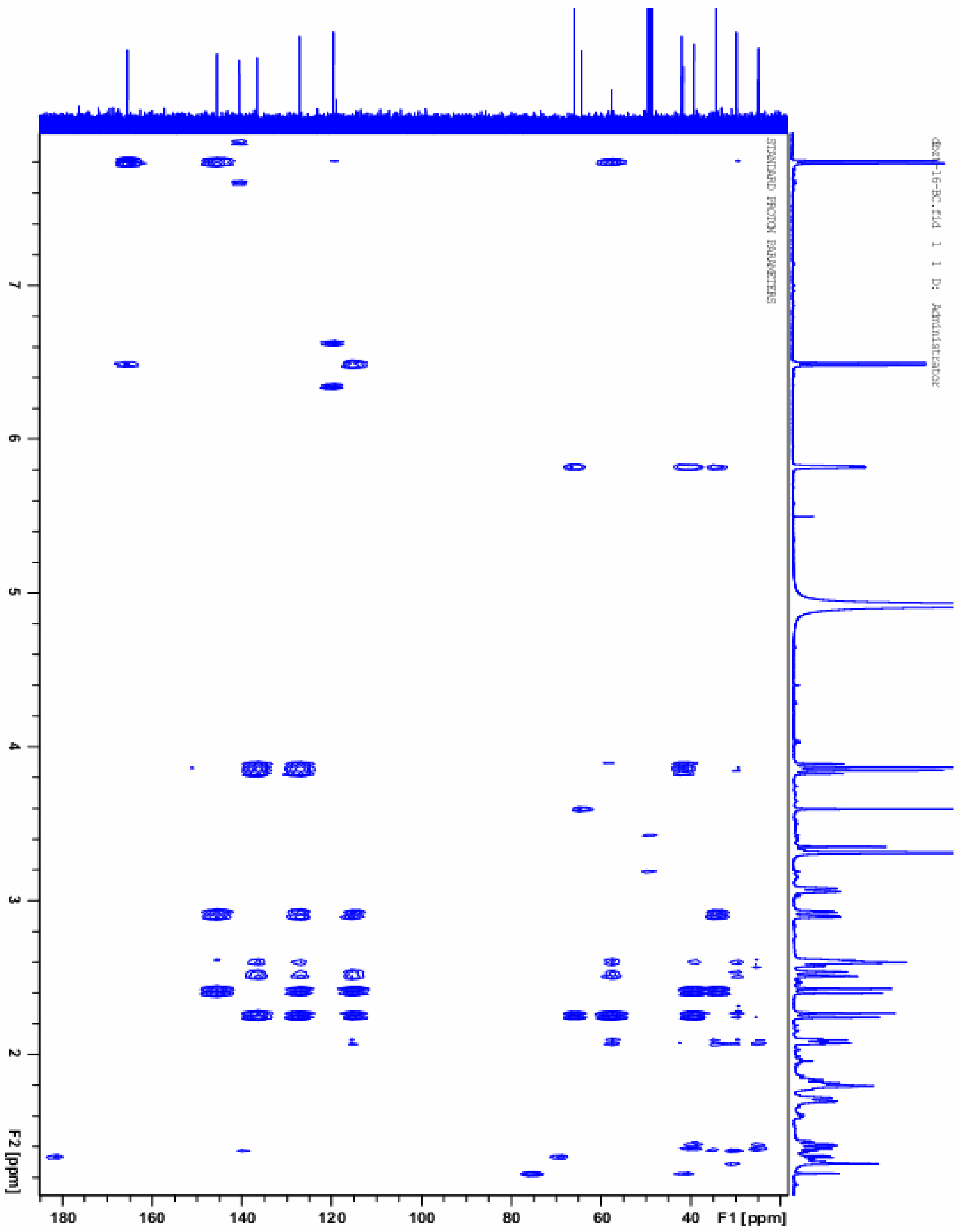

**Figure S6.** HRESIMS spectrum of compound **1**.

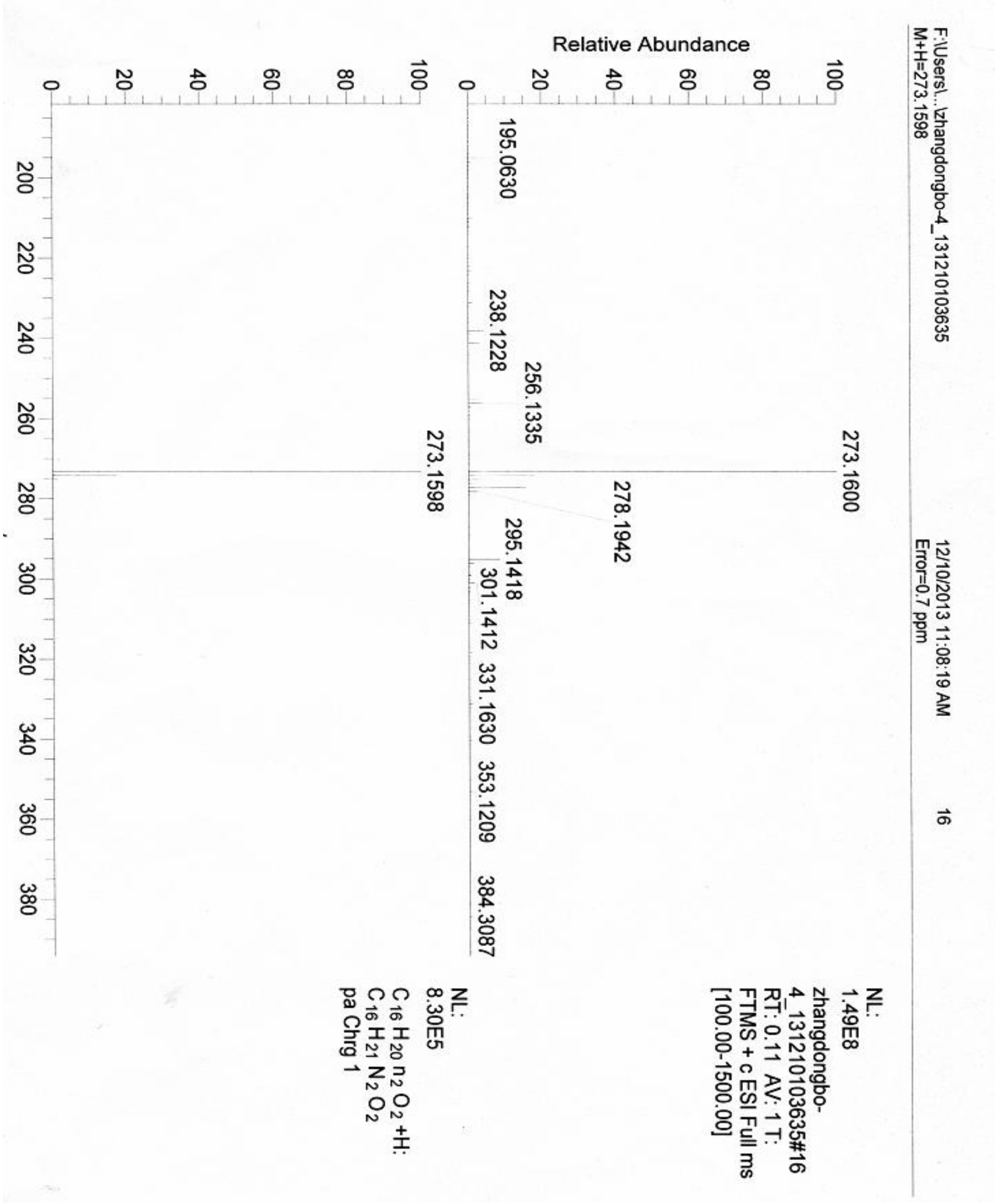

**Figure S7.** $^1$H-NMR spectrum of compound **2**.

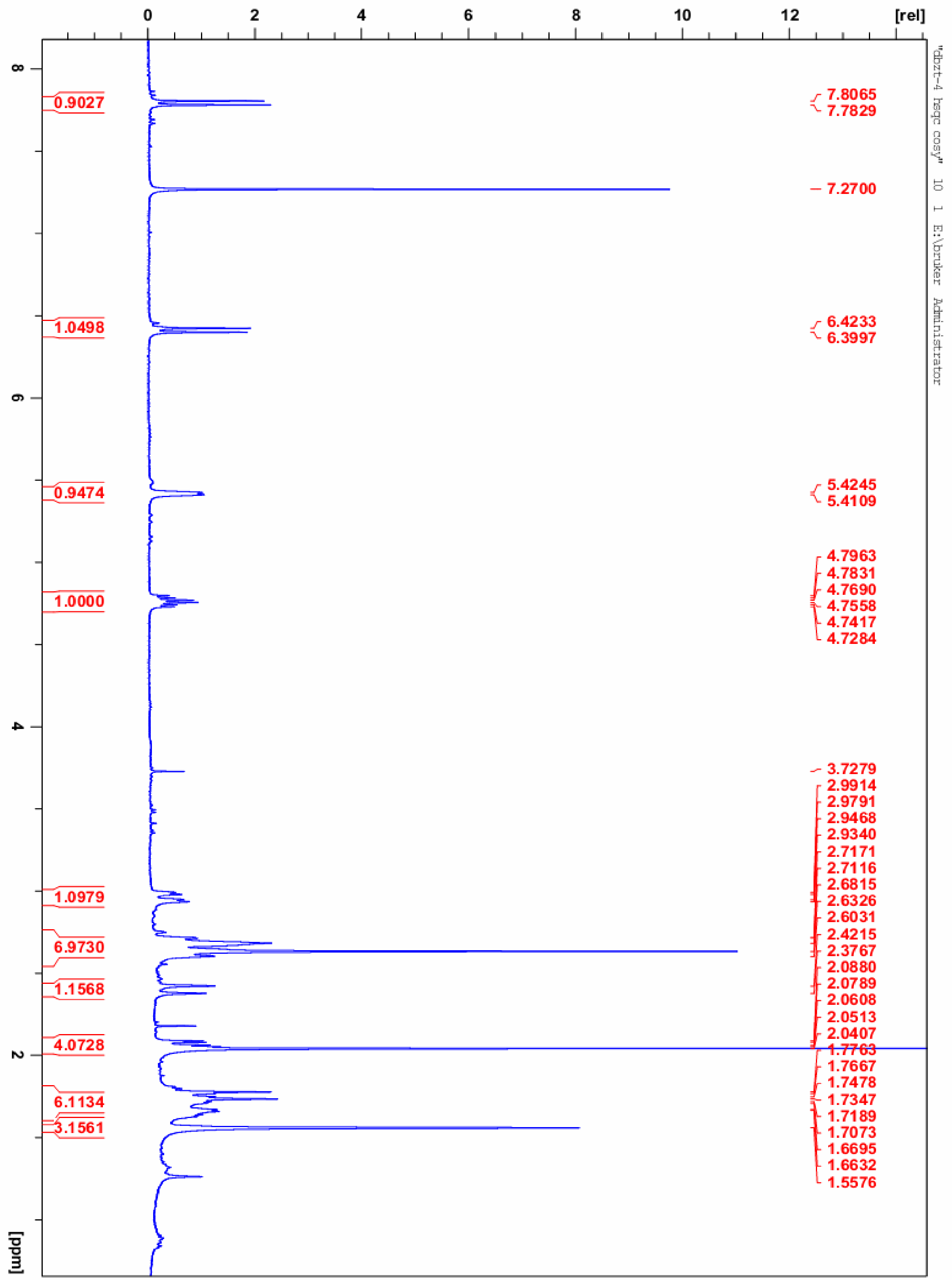

**Figure S8.** $^{13}$C-NMR spectrum of compound **2**.

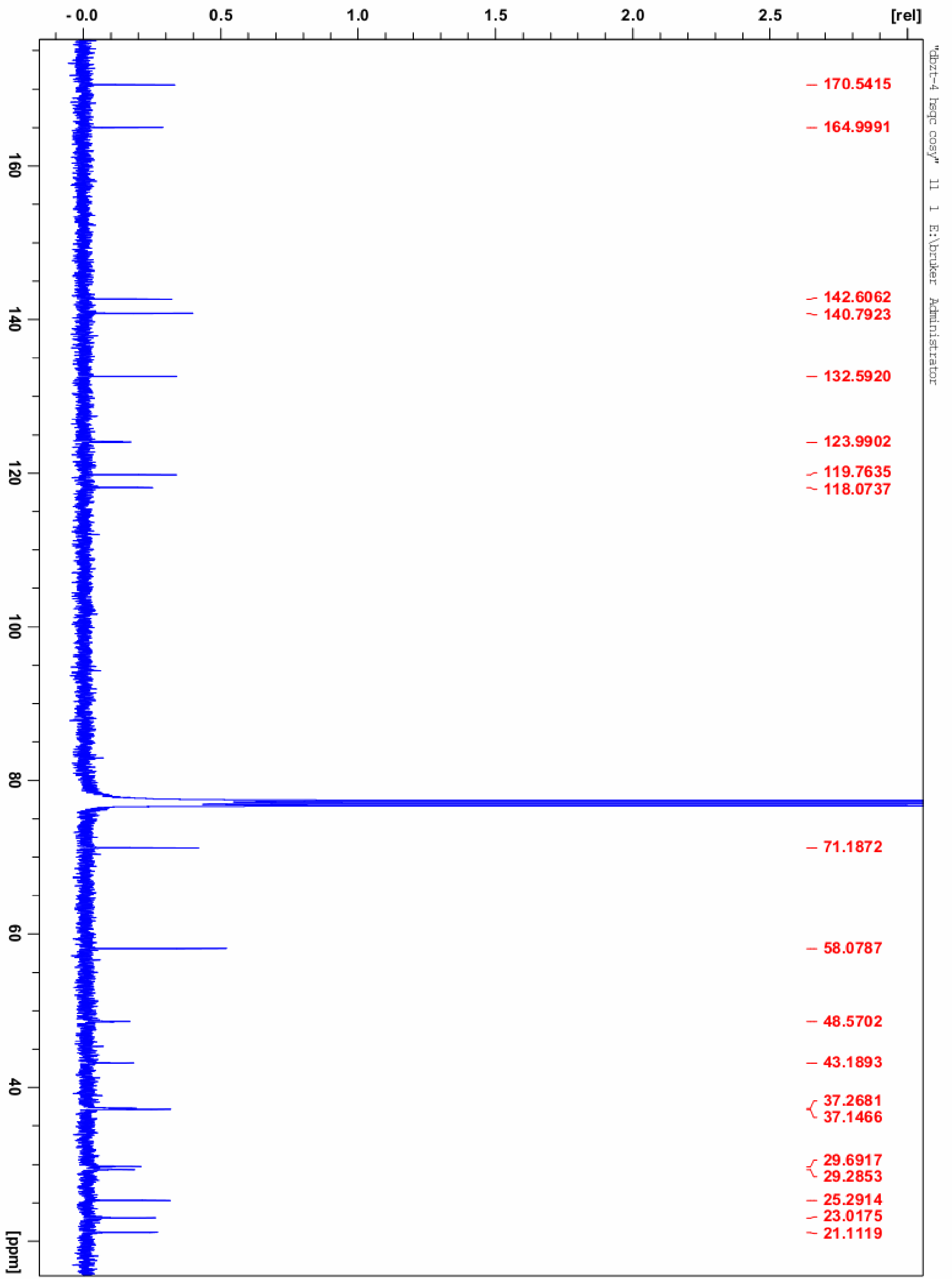

**Figure S9.** $^{1}H$-$^{1}H$ COSY spectrum of compound **2**.

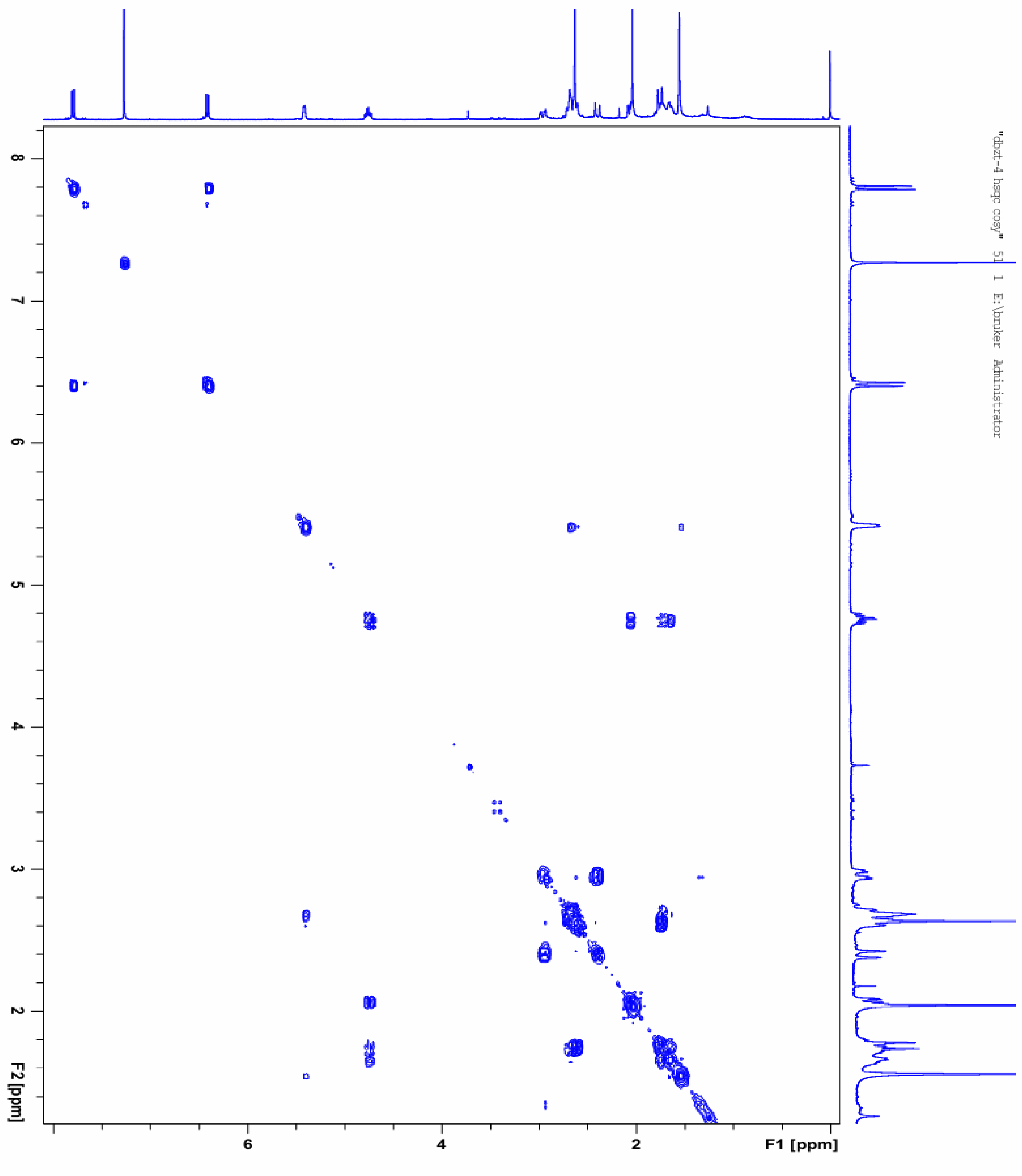

**Figure S10.** HSQC spectrum of compound **2**.

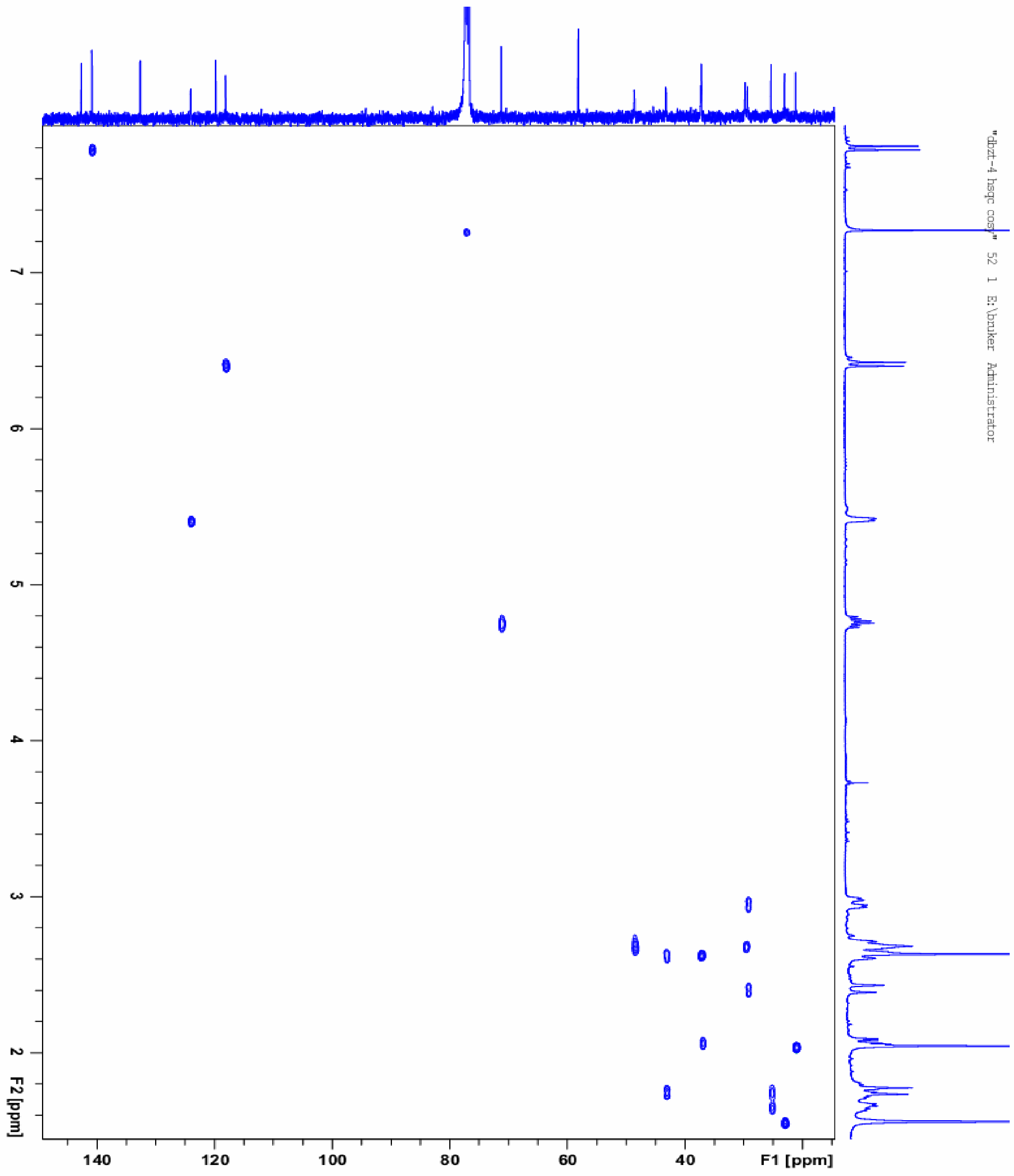

**Figure S11.** HMBC spectrum of compound **2**.

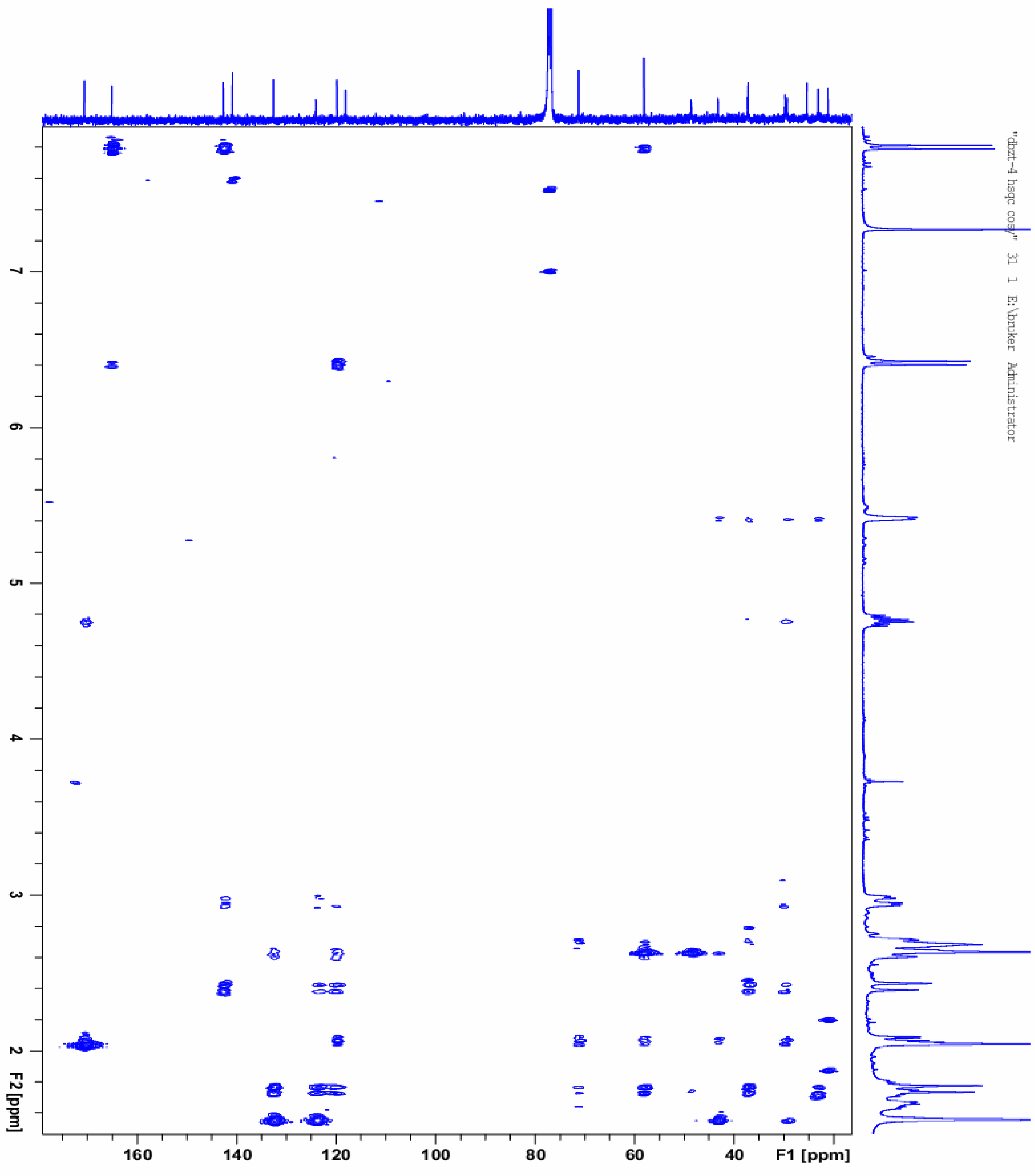

**Figure S12.** HRESIMS spectrum of compound **2**.

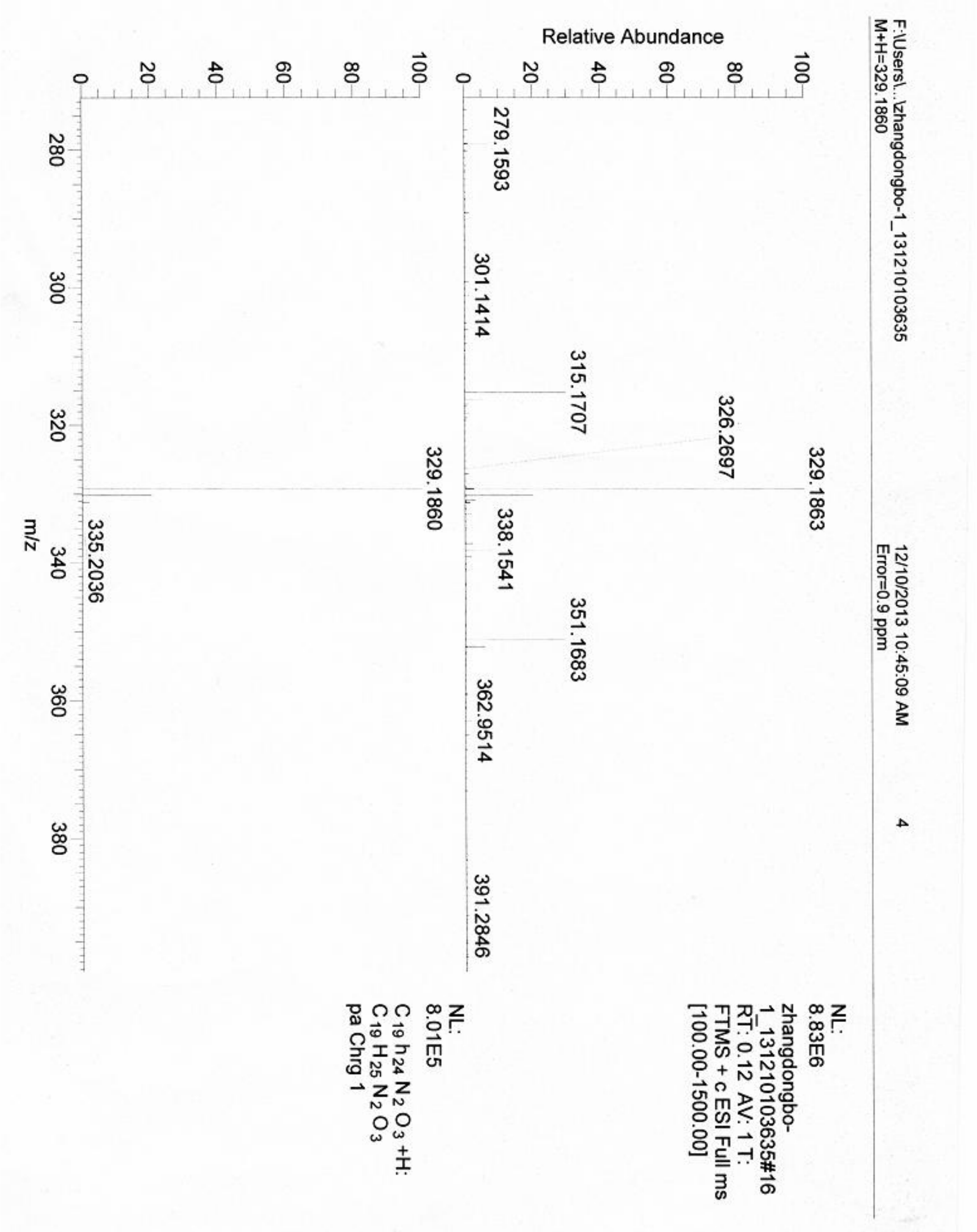

**Figure S13.** $^{1}$H-NMR spectrum of compound **3**.

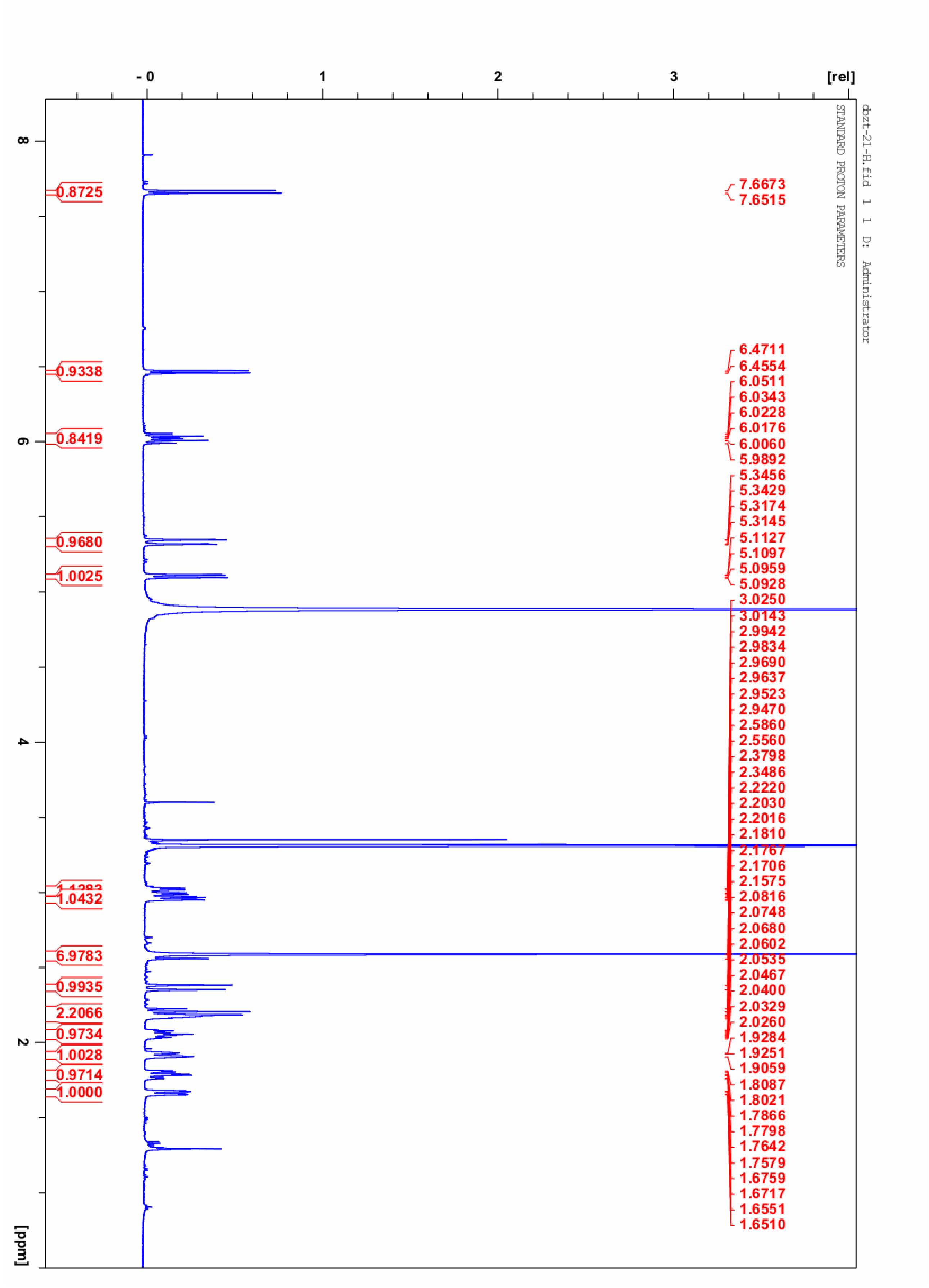

**Figure S14.** $^{13}$C-NMR spectrum of compound **3**.

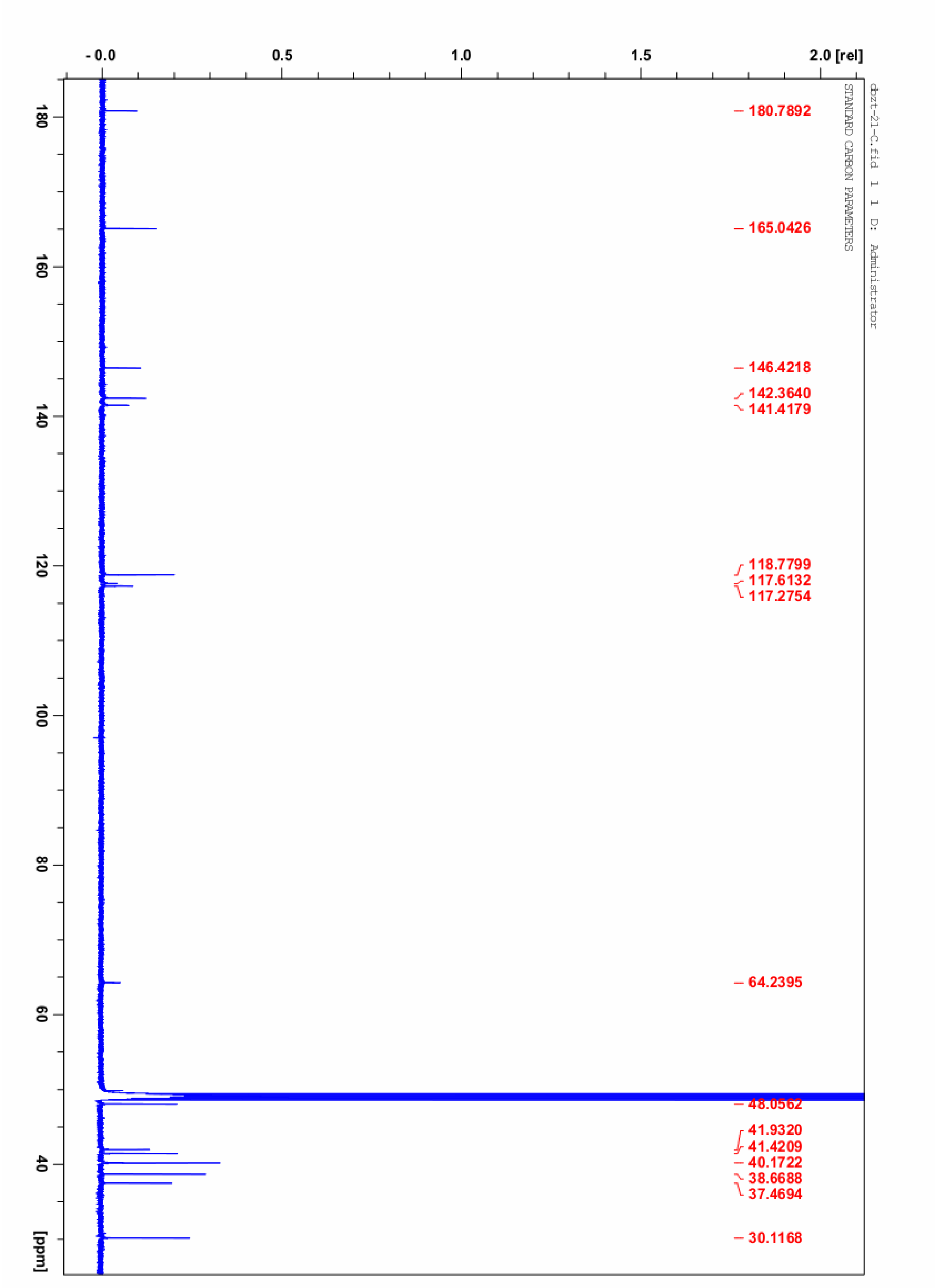

**Figure S15.** $^{1}H$-$^{1}H$ COSY spectrum of compound **3**.

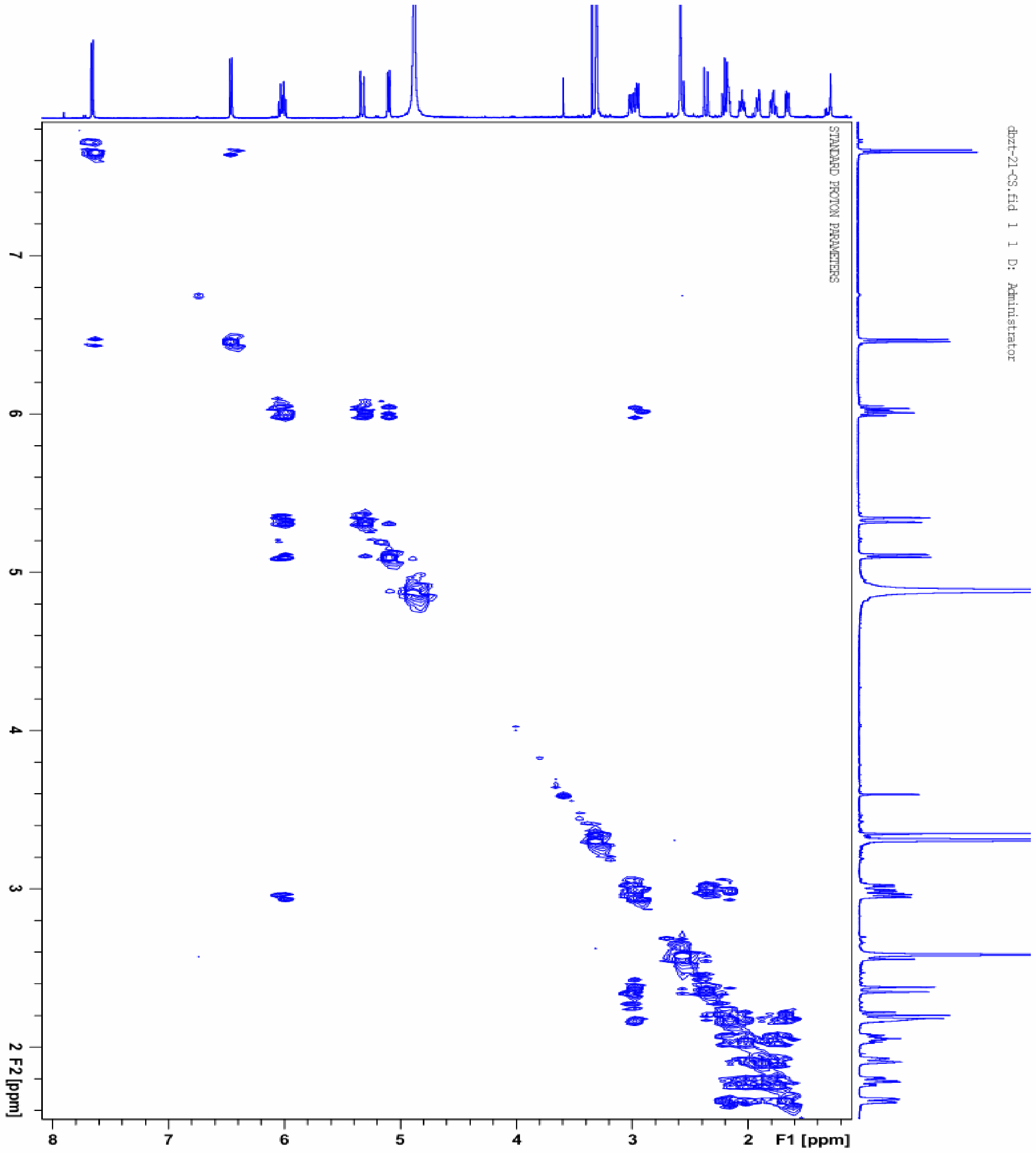

**Figure S16.** HSQC spectrum of compound **3**.

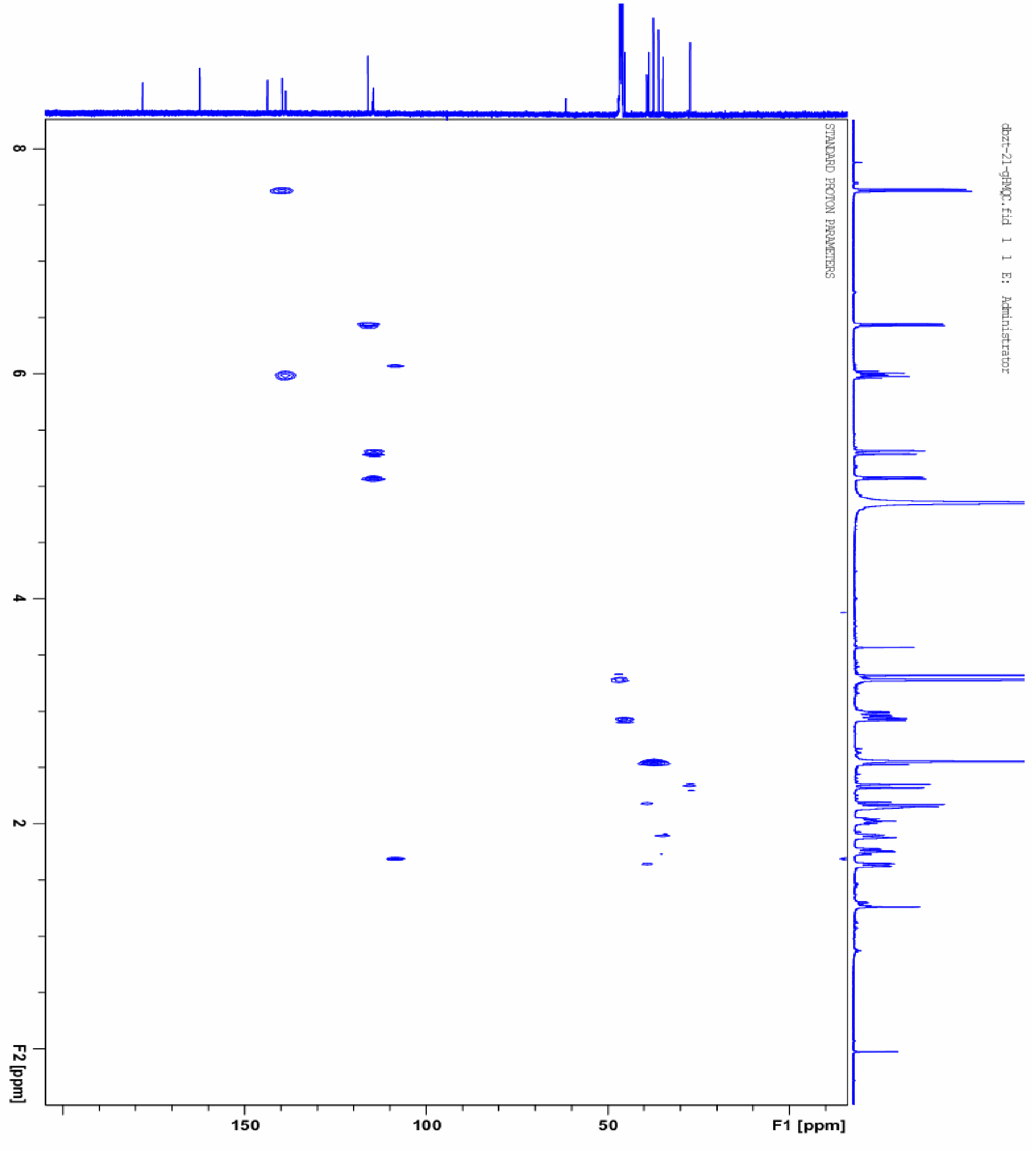

**Figure S17.** HMBC spectrum of compound **3**.

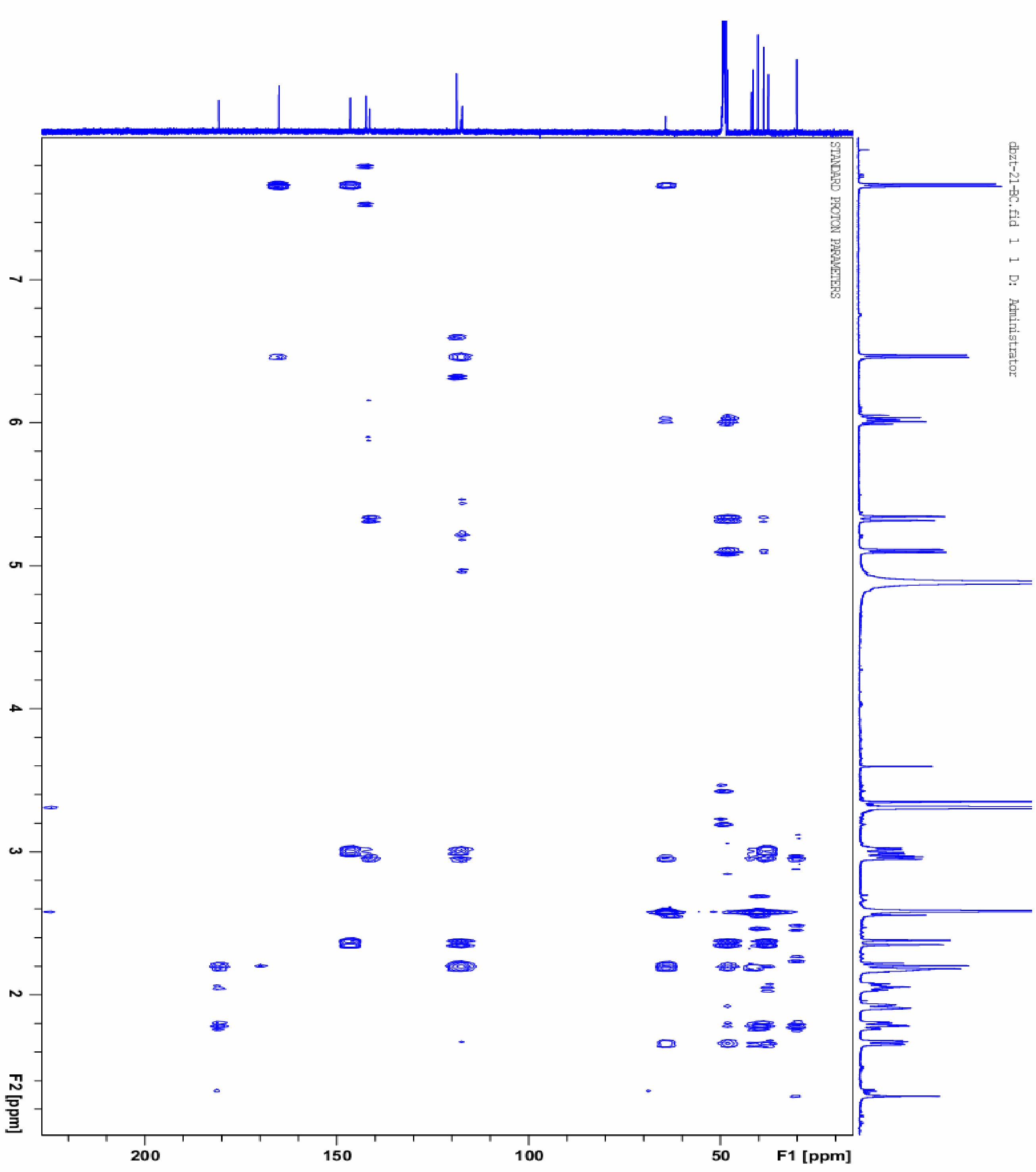

**Figure S18.** HRESIMS spectrum of compound **3**.

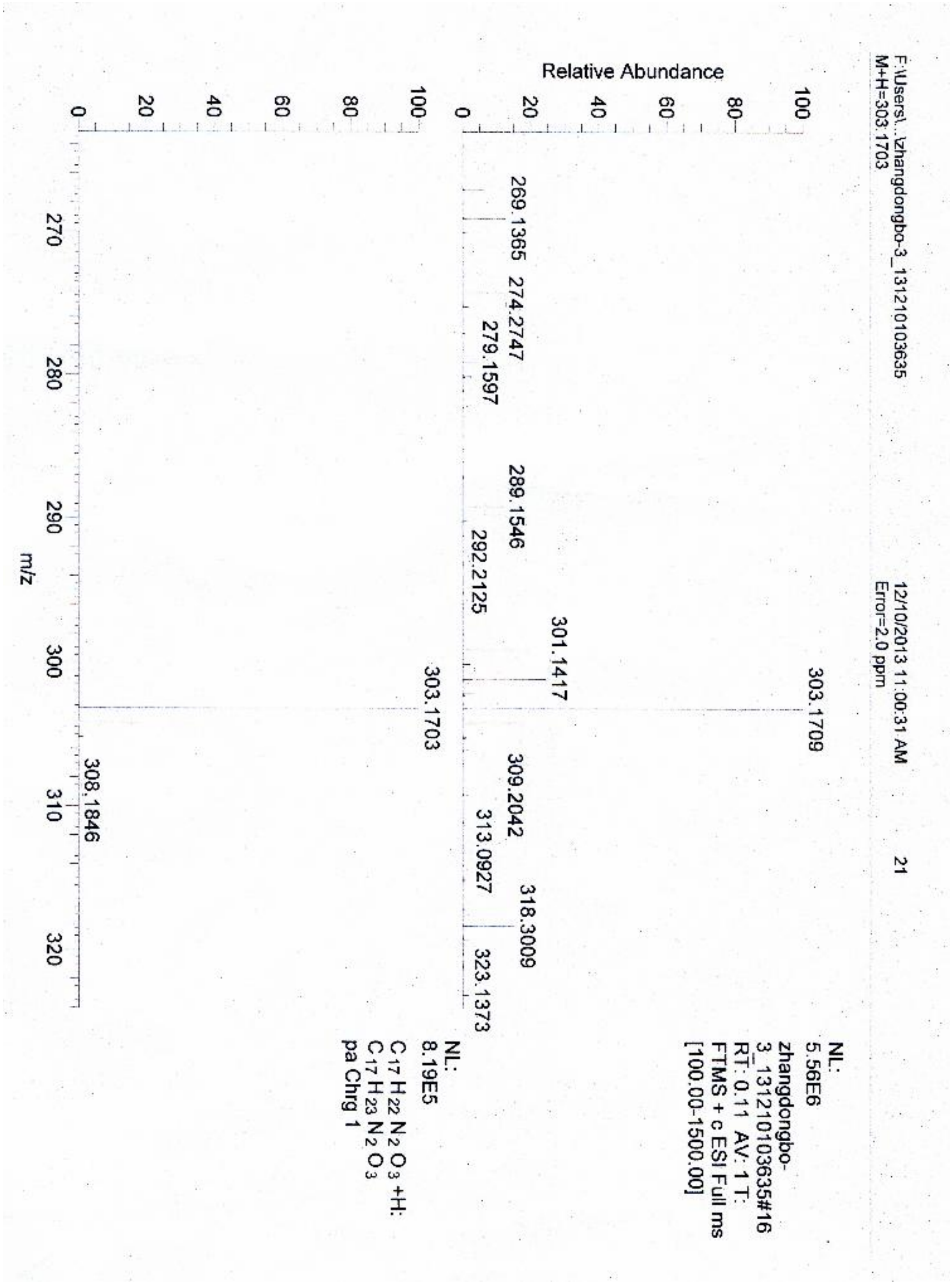

**Figure S19.** $^{1}$H-NMR spectrum of compound **4**.

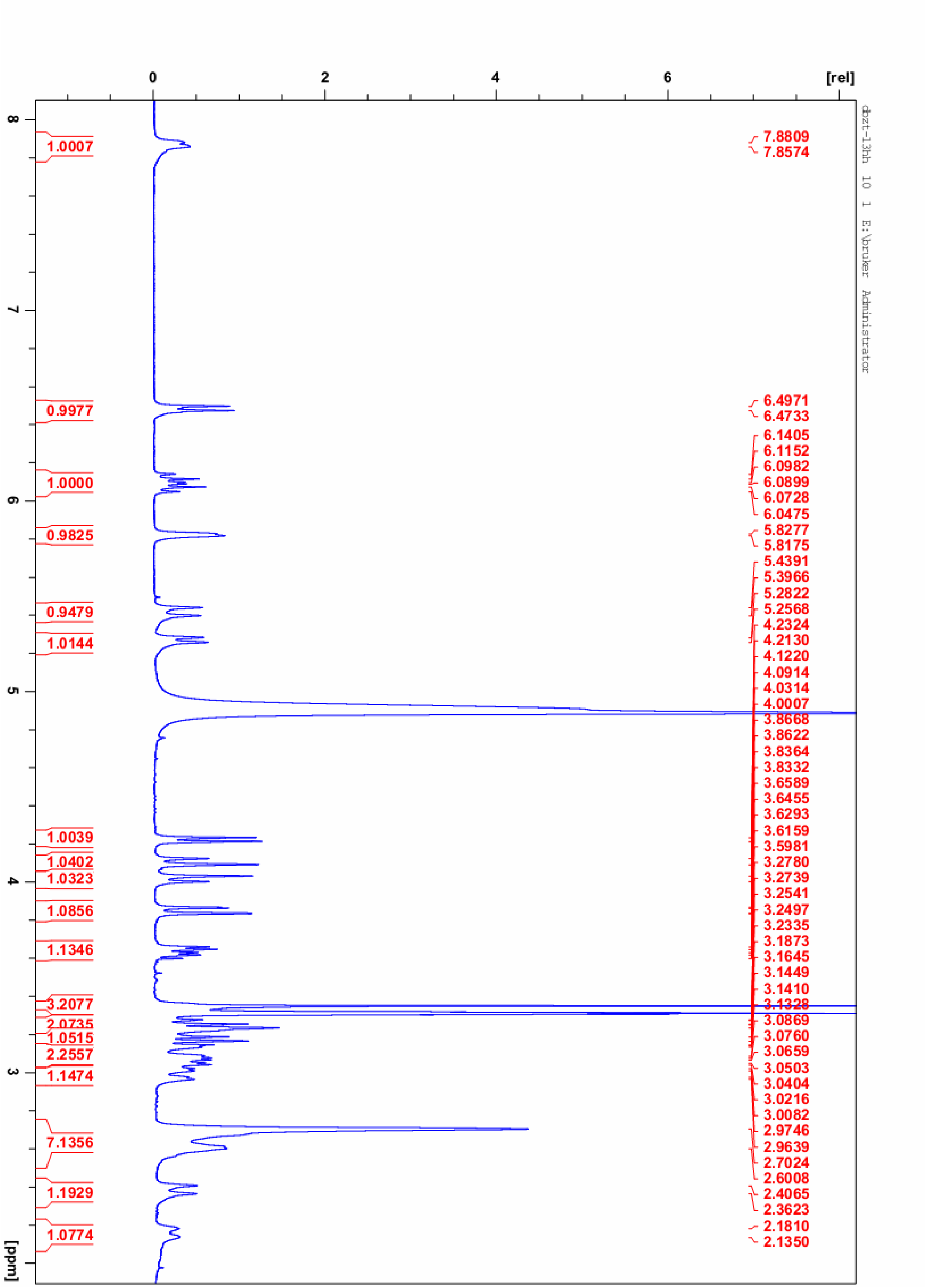

**Figure S20.** $^{13}$C-NMR spectrum of compound **4**.

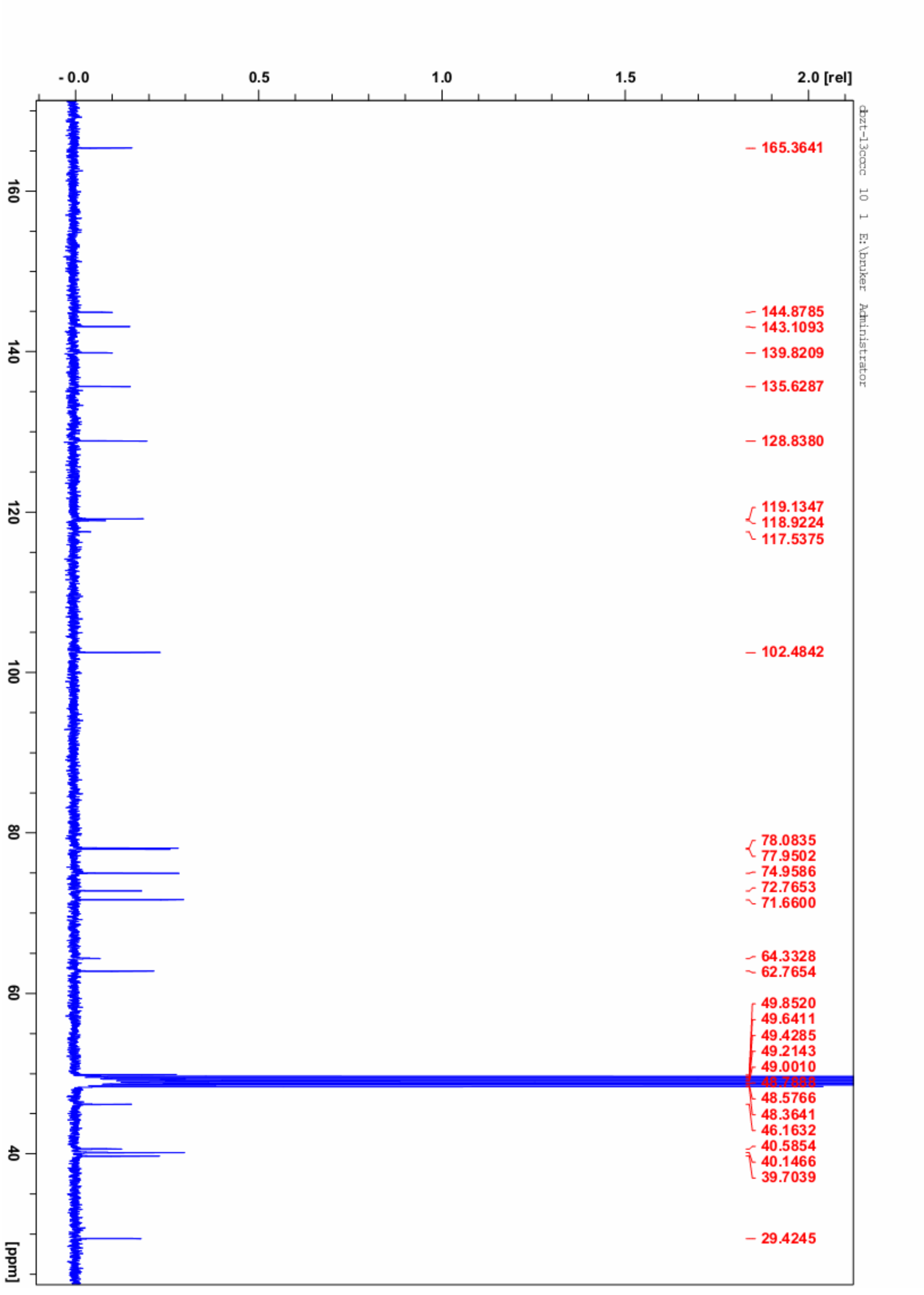

**Figure S21.** $^{1}$H-$^{1}$H COSY spectrum of compound **4**.

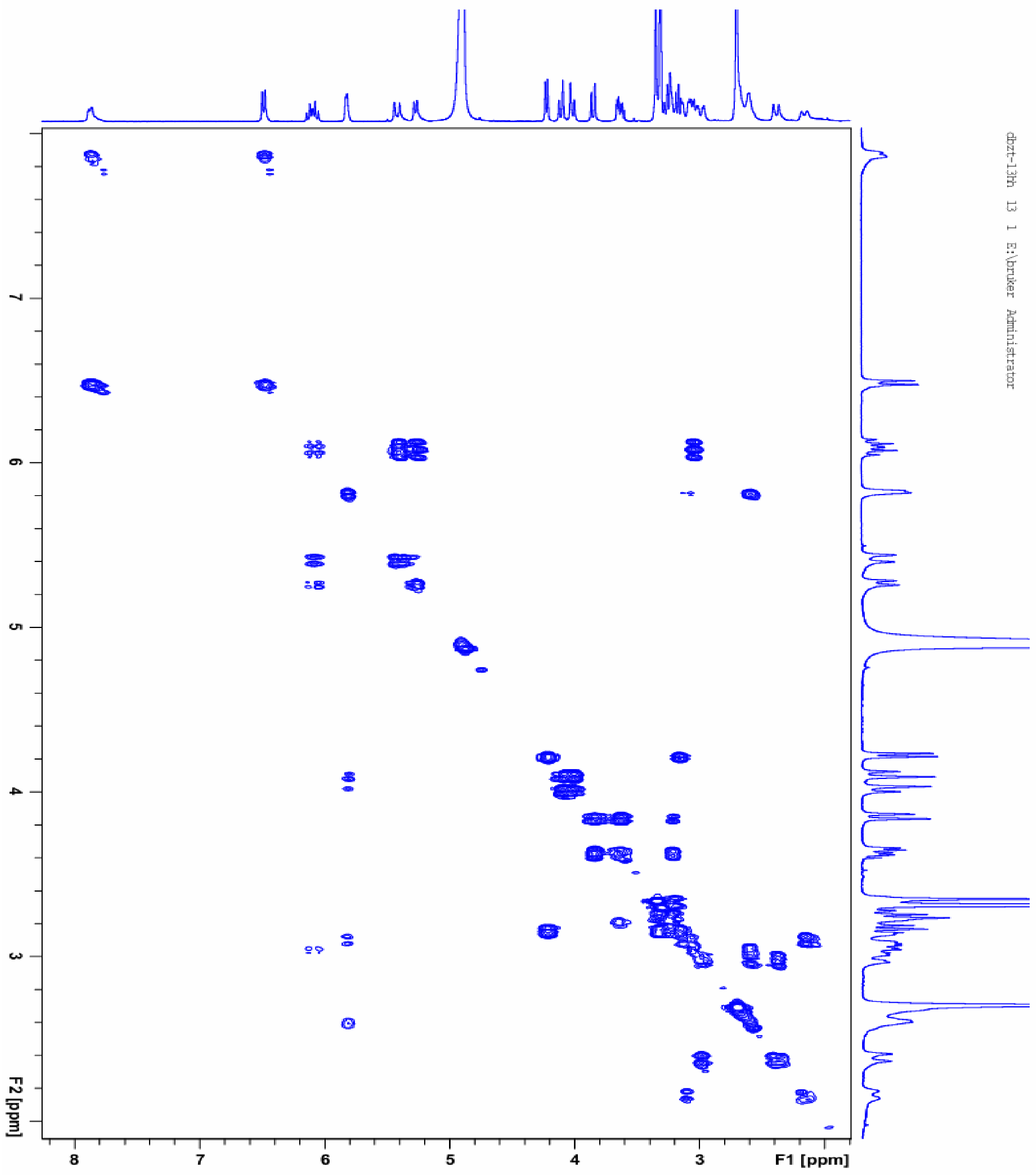

**Figure S22.** HSQC spectrum of compound **4**.

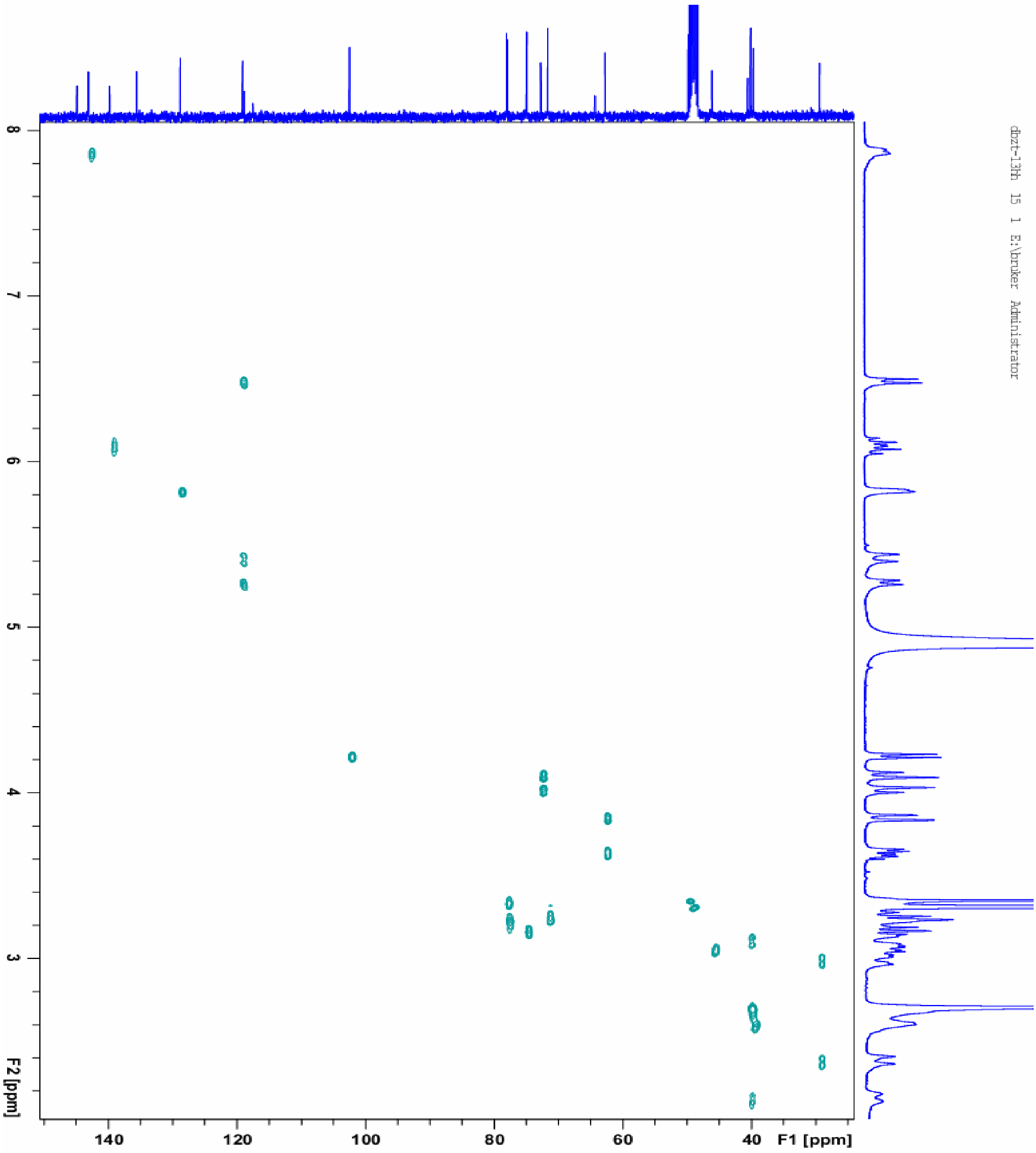

**Figure S23.** HMBC spectrum of compound **4**.

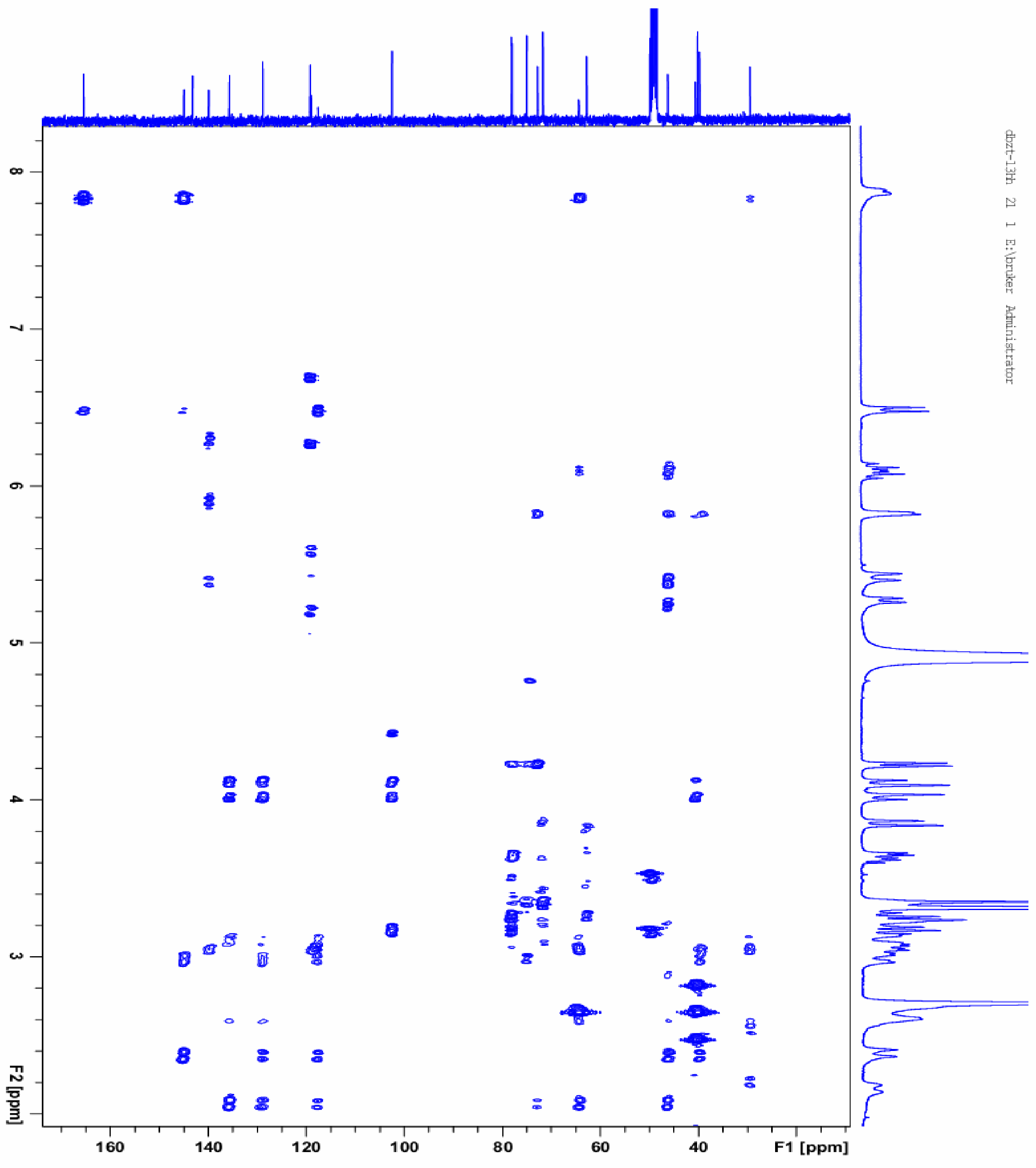

**Figure S24.** HRESIMS spectrum of compound **4**.

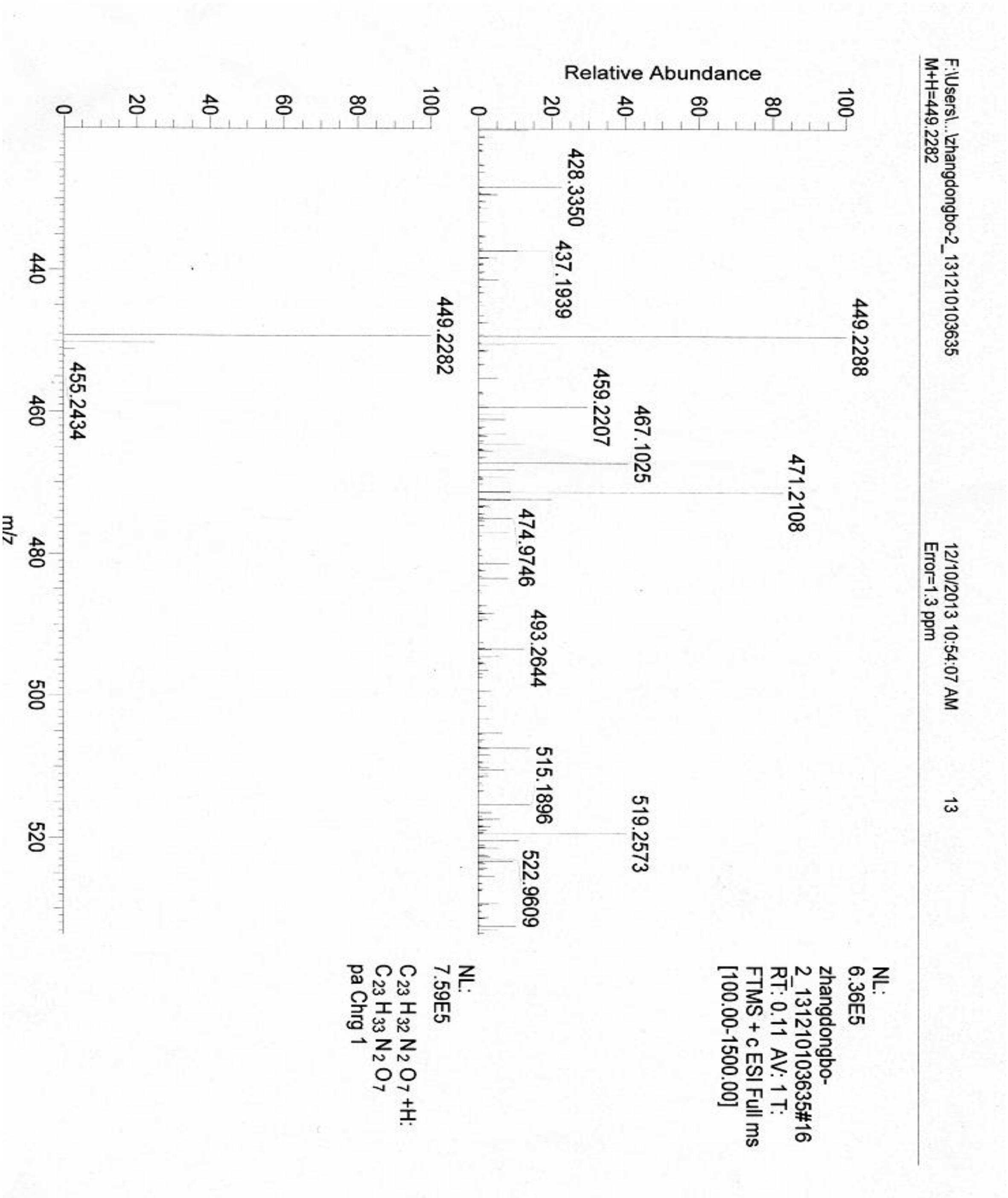

Supplement: Supplementary file 1 [file molecules-19-09999-s001.pdf]
